# Scale free avalanches in excitatory-inhibitory populations of spiking neurons with conductance based synaptic currents

**DOI:** 10.1007/s10827-022-00838-4

**Published:** 2022-10-25

**Authors:** Masud Ehsani, Jürgen Jost

**Affiliations:** 1 Max Planck Institute for Mathematics in Sciences, Inselstr.22, Leipzig, 04103 Saxony Germany; 2 Santa Fe Institute, 1399 Hyde Park Rd, Santa Fe, NM 87501 United States

**Keywords:** Critical brain hypothesis, Scale free avalanches, Linear poisson neuron, Bogdanov-takens bifurcation

## Abstract

We investigate spontaneous critical dynamics of excitatory and inhibitory (EI) sparsely connected populations of spiking leaky integrate-and-fire neurons with conductance-based synapses. We use a bottom-up approach to derive a single neuron gain function and a linear Poisson neuron approximation which we use to study mean-field dynamics of the EI population and its bifurcations. In the low firing rate regime, the quiescent state loses stability due to saddle-node or Hopf bifurcations. In particular, at the Bogdanov-Takens (BT) bifurcation point which is the intersection of the Hopf bifurcation and the saddle-node bifurcation lines of the 2D dynamical system, the network shows avalanche dynamics with power-law avalanche size and duration distributions. This matches the characteristics of low firing spontaneous activity in the cortex. By linearizing gain functions and excitatory and inhibitory nullclines, we can approximate the location of the BT bifurcation point. This point in the control parameter phase space corresponds to the internal balance of excitation and inhibition and a slight excess of external excitatory input to the excitatory population. Due to the tight balance of average excitation and inhibition currents, the firing of the individual cells is fluctuation-driven. Around the BT point, the spiking of neurons is a Poisson process and the population average membrane potential of neurons is approximately at the middle of the operating interval $[V_{Rest}, V_{th}]$. Moreover, the EI network is close to both oscillatory and active-inactive phase transition regimes.

**Supplementary Information:**

The online version contains supplementary material available at 10.1007/s10827-022-00838-4.

## Introduction

Experiments have shown that in the absence of stimuli, the cortical population of neurons shows rich dynamical patterns, called spontaneous activity, which do not look random and entirely noise-driven but are structured in spatiotemporal patterns (Takeda et al. (2016); Thompson et al. (2014)). Spontaneous activity is assumed to be the substrate or background state of the neural system with functional significance (Raichle (2010)). Experimental findings on different temporal and spatial resolutions highlight the scale-free characteristic of spontaneous activity.

In microcircuits of the brain during spontaneous activity, we observe avalanche dynamics. This mode of activity was first closely investigated by Beggs and Plenz (2003) in cultured slices of rat cortex using a multi-electrode array with an inter-electrode distance of $200 \mu m$ to record local field potentials (LFP). An avalanche is defined as almost synchronized epochs of activity separated by usually long periods of inactivity. At higher temporal resolution this seemingly synchronized pattern appears as a cascade of activity in micro-electrodes arrays initiated from one (or a few) local sites that propagate through the network and finally terminate. The main finding of this seminal experimental paper is power-law scaling of the probability density function for size and duration of avalanches. And causing an excitation and inhibition imbalance by injecting specific drugs destroys power-law scaling. Further studies confirm these results in different setups like awake monkeys (Petermann et al. (2009)), in the cerebral cortex and hippocampus of anesthetized, asleep, and awake rats (Ribeiro et al. (2010)) and the visual cortex of an anesthetized cat (Hahn et al. (2010)). Besides LFP data several studies report the scale-free avalanche size distribution based on spike data (Friedman et al. (2012), Hahn et al. (2010), Mazzoni et al. (2007)). Friedman et al. (2012) analyzed cultured slices of cortical tissue and collected data at individual neurons with different spacing. Klaus et al. (2011) showed that a power law is the best fit for neural avalanches collected from in vivo and in vitro experiments.

Besides power-law scaling of size and duration of avalanches with exponents $\tau \sim -1.5$ and $\alpha \sim -2$, respectively, they showed that the temporal profile of avalanches is described by a single universal scaling function. Average size versus average duration of avalanches is also a power-law with $\langle s\rangle = \langle T\rangle ^{\dfrac{1}{\sigma \nu z}}$ linked by a scaling relation $\dfrac{\alpha -1}{\tau -1} = \dfrac{1}{\sigma \nu z}$ between exponents. In addition, the mean temporal profile of avalanches follows a scaling form as in non-equilibrium critical dynamics,

1 $$\begin{aligned} S(t,T) \sim T^{1/\sigma \nu z -1} F(t/T) \end{aligned}$$

Data sets collapse to the scaling function very well. The appearance of power laws, scaling relations among their exponents, data collapse, and sensitivity to the imbalance of excitation and inhibition led to the hypothesis that somehow the brain is poised near criticality by a self-organization mechanism with the balance of excitatory and inhibitory rates as the self-organizing parameter. In this direction, many models have been presented in the past decades. Short-term plasticity in excitatory neuronal models has been investigated as a self-organizing principle for a non-conservative neuronal model Levina et al. (2009, 2007); Peng and Beggs (2013); di Santo et al. (2018); Brochini et al. (2016). In addition, self-organization by other control parameters like degree of connectivity or synaptic strength Bornholdt and Roehl (2003); Rybarsch and Bornholdt (2014), STDP Meisel and Gross (2009), and balanced input Benayoun et al. (2010) has been studied. Cowan et al. (2013) used the method of path integral representation in the stochastic model of spiking neurons supplemented by anti-Hebbian synaptic plasticity as the self-organizing mechanism. Their network possesses bistability close to the saddle-node bifurcation point which is the origin of the avalanche behavior in the system.

On the other hand, the spontaneous firing of single neocortical neurons is considered to be a noisy, stochastic process resembling a Poisson point process. It has been claimed that the balance of excitation and inhibition is a necessary condition for the noisy irregular firing of individual neurons as well as scale free avalanche patterns at the population level. Since the network is settled in a balanced state, a small deviation in the balance condition leads to a local change in the firing rate. Therefore, the system is highly sensitive to input while maintaining a low firing rate and highly variable spike trains at the individual neuron level. Inhibitory-excitatory balance can lead to asynchronous cortical states in local populations (Brunel and Hakim (1999, 2000, 2008)) and the emergence of waves and fronts at a larger scale of cortical activity (Ermentrout (1998); Bressloff (2011)). At the level of individual neurons, this balance leads to highly irregular firing of neurons with inter-spike interval distribution with CV close to one and thus resembling a Poisson process (Softky and Koch (1993)). Studies using the voltage clamp method tracking conductance of excitatory and inhibitory synapses on neurons both in vivo and in vitro, confirmed that there exist proportionality and balance of inhibitory and excitatory currents during upstate (Haider et al. (2006)), sensory input (Shu et al. (2003)) and spontaneous activity (Okun and Lampl (2008)). In Karimipanah et al. (2017), authors showed irregular spiking with coefficient of variation (CV) of inter-spike intervals greater than one occurs at the edge of criticality in a binary probabilistic network of expiatory neurons.

Most of the models discussed above studied critical avalanches in only expiatory population. In an Inh.-Exc. Network, Benayoun et al. proposed a stochastic model of spiking neurons which matches the Wilson-Cowan mean field in the limit of infinite system size that shows scale-free avalanches in the balanced state in which the sum of excitation and inhibition is much larger than the net difference between them (Benayoun et al. (2010)). Under symmetry conditions on weights, the Jacobian has negative eigenvalues close to zero in the balanced state suggesting the system operating in the vicinity of a Bogdanov-Takens bifurcation point. In this model, the Poisson firing of the neurons is presumed, and symmetric synaptic connections and $O(N^{-1})$ scaling of weights is required for applying the linear noise approximation. Furthermore, the origin of the scale-free behavior and the bifurcation diagram of the model in a wider regime of parameters has not been studied and the power law exponents in their model do not match the experimental results. Girardi-Schappo et al. (2020) reported scale-free avalanches in fluctuation driven asynchronous irregular (AI) state of firing in discrete-time stochastic integrate-and fire Exc.- Inh. Neuronal population. Source of noise in their model is as well intrinsic stochastic of neurons and the all-to-all connectivity requires $O(N^{-1})$ scaling of weights for the system to show balanced currents. Carvalho et al. (2021), investigate how sub sampling from the EI critical model of Girardi-Schappo , near active inactive phase transition matches deviation form power law exponents observed in experimental study (Criticality between Cortical States, Fontenele et al. (2019)).

In this work, we start from a bottom-up approach by analytically investigating conditions on Poisson firing at the single neuron level and introducing conditions on the balance of inhibitory and excitatory currents. Poisson firing condition has been studied before in integrate and fire neurons with non-conductance based and only excitatory current (Kistler and van Hemmen (2000)). Here, we investigate conditions on balanced conductance-based input currents that lead to Possion firing. Next, we build a linear Poisson neuron model with minimal error in the low firing rate regime. The linear Poisson regime of firing is a segment of the dynamical regime of the neuron response. We can use this linearization to form an approximate linearized gain function. This gain function can then be used to investigate dynamics of a sparsely or all to all connected homogeneous network of inhibitory and excitatory neurons and its bifurcation diagram. We also introduce another compatible approximation of gain functions by sigmoids. We observe avalanche patterns with power-law distributed sizes and duration at the intersection of saddle-node and Hopf bifurcation lines, i.e., at a Bogdanov-Takens (BT) bifurcation point of the mean field equations. At this state, the volume of the basin of attraction of the quiescent state is small, and internal noise can make the system escape from it. Activity grows and decays back to the quiescent state along heteroclinic orbits connecting the two saddle points in the low firing rate regime which coincides with the slow manifold of the fixed points. Along this slow manifold, there is a tight temporal balance of excitation and inhibition in the forms of avalanches of highly variable sizes. The balance of excitatory and inhibitory inputs leads to stationary values of membrane potentials that allow Poisson firing at the single neuron level and avalanche type dynamics at the population level. The firing of neurons is due to the accumulation of internal currents, and external input by itself does not suffice to trigger firing. However, external input imbalance to excitatory and inhibitory populations is needed for the initiation of the avalanche. During each avalanche at the BT point, each neuron on average activates one another neuron which leads to termination of avalanches with power-law distributed durations and sizes. This is the case when currents to single cells are balanced in a way that excess excitation firing is compensated by inhibitory feedback. A linear relation between excitatory and inhibitory rates close to the BT point enables us to write down the dynamics of the excitatory population as a branching process. Close to the BT point the branching parameter is close to one which is indicative of the critical state.

Tuning the system at the BT point can be attained by the balance of inhibitory feedback leading to a condition on synaptic weights and adjustment of excess external drive to the excitatory population. This is investigated in another article (Ehsani and Jost (2022)), where we show how learning by STDP and homeostatic synaptic plasticity as self-organizing principles can tune the system close to the BT point by regulating the inhibitory feedback strength and excitatory population gain.

## Neuron model and network architecture

We use an integrate and fire neuron model in which the change in the membrane voltage of the neuron receiving time dependent synaptic current *i*(*t*) follows :

2 $$\begin{aligned} C \dfrac{d v(t)}{dt} = g_{Leak}( v_{Leak} - v(t)) + i(t), \end{aligned}$$

for $v(t)<v_{th}$ . When the membrane voltage reaches $v_{th} = -50mv$, the neuron spikes and immediately its membrane voltage resets to $v_{rest}$ which is equal to $v_{Leak} = -65mv$.

In the following, we want to concentrate on a model with just one type of inhibitory and one type of excitatory synapses, which can be seen as the average effect of the two types of synapses. We can write the synaptic inhibitory and excitatory current as

3 $$\begin{aligned} i(t) = g_{inh}(t) * ( V_{Rinh} - v(t)) + g_{exc}(t) * (V_{Rexc} - v(t)) \end{aligned}$$

$V_{Rinh}$ and $V_{Rexc}$ are the reverse potentials of excitatory and inhibitory ion channels, and based on experimental studies we choose values of $-80mv$ and 0*mv* for them respectively. $g_{inh}(t)$ and $g_{exc}(t)$ are the conductances of inhibitory and excitatory ion channels. These conductances are changing by the inhibitory and excitatory input to the cell. Each spike of a presynaptic inhibitory or excitatory neuron *j* to a postsynaptic neuron *k* that is received by *k* at time $t_0$ will change the inhibitory or excitatory ion channel conductance of the postsynaptic neuron for $t>t_0$ according to

4 $$\begin{aligned} \begin{aligned} g_{Inh}^k(t)&= w_{kj} * g_{0}^{inh} * exp(-\dfrac{t-t_0}{\tau _{syn}^{inh}}) \\ g_{Exc}^k(t)&= w_{kj} * g_{0}^{exc} * exp(-\dfrac{t-t_0}{\tau _{syn}^{exc}}) \end{aligned} \end{aligned}$$

Here we assume that the rise time of synaptic conductances is very small compared to other time scales in the model and therefore, we modeled the synaptic current by a decay term with synaptic decay time constant $\tau _{syn}$ which we assume to be the same value of 5*ms* for both inhibitory and excitatory synapses. In the remainder of this work, in the simulation, we consider a population of $N_{Exc} =2*10^4$ and $N_{Inh} = 0.25*N_{Exc}$ inhibitory spiking neurons with conductance-based currents introduced in this section. Each excitatory neuron in the population is randomly connected to $k_{EE} = \dfrac{N_{Exc}}{100}=200$ excitatory and $k_{EI} = \dfrac{k_{EE}}{4}$ inhibitory neurons and each inhibitory neuron is connected to $k_{IE} = k_{EE}$ and $k_{II} = \dfrac{k_{EE}}{4}$ excitatory and inhibitory neurons, respectively. The weights of excitatory synaptic connections are in a range that $10-20$ synchronous excitatory spikes suffice to depolarize the target neuron to the level of its firing threshold when it is initially at rest at the time of input arrival. Weights are being drawn from a log-normal probability density with low variance. Therefore, approximately $O(\sqrt{k_{EE}})$ spikes are adequate for firing. Assuming homogeneity in the population as we have discussed in the introduction we can build a mean-field equation for the excitatory and inhibitory population in this sparse network, assuming each neuron receives input with the same statistics.

## Results

### Response of a single neuron to the Poisson input

In this section, we want to consider the response of the neuron to a specific type of current, namely Poisson input. The reason to consider this type of input is that in an asynchronous firing state neurons receive Poisson input from other neurons. Assume that the number of afferents to each neuron is high and the population activity is nearly constant with firing rate *r*. Assuming homogeneity in the number of connections and weights, then at any moment the probability distribution function that for a neuron, *k* presynaptic neurons out of a total number of *n* presynaptic neurons are active is a binomial $$f(n,k,r)= \begin{bmatrix} n\\ k \\ \end{bmatrix} r^k(1-r)^{n-k}$$ which in the regime $r<<1$ and large *n* is well approximated by a Poisson distribution with parameter *nr*.

We first study the response of the neuron to a non-fluctuating constant periodic synaptic current. Suppose the target neuron receives constant numbers $\rho _E$ and $\rho _I$ of excitatory and inhibitory spikes per unit time, with all the excitatory spikes having the same strength $w_E$ and all the inhibitory spikes having the strength $w_I$. The conductance of the excitatory channels $g_{exc}(t)$ is modified by excitatory spikes arriving at times $s<t$ :

5 $$\begin{aligned} g_{exc}(t) = \int _{-\infty }^{t} g_{exc}^0 w_E \rho _E exp(-\dfrac{t-s}{\tau _{syn}^{exc}}) ds = g_{exc}^0 w_E \rho _E \tau _{syn}^{exc} \end{aligned}$$

The same formula applies for the constant inhibitory current. The potential of the target neuron fed by this current will reach a stationary value. If this stationary limit is greater than $V_{th}$ then the target neuron will fire periodically. This constraint reads as :

6 $$\begin{aligned} \rho _I < \dfrac{g_{leak}*(V_{th} - V_{rest}) + g_{exc}^0*w_E*\rho _E*\tau * V_{th}}{g_{inh}^0*w_I*\tau (V_{inh} - V_{th}) } \end{aligned}$$

The stationary limit of the potential is a weighted average of reverse potentials,

7 $$\begin{aligned} V_{st} = \dfrac{g_{L}V_{L} + g_{exc}^0 w_E \rho _E \tau V_{Rexc} + g_{inh}^0 w_I \rho _I \tau V_{Rinh} }{g_{L} + g_{exc}^0 w_E \rho _E \tau + g_{inh}^0 w_I \rho _I \tau } \end{aligned}$$

If input rates satisfy Eq. 6, the output firing rate will be

8 $$\begin{aligned} \rho _{out} = (g_{leak} + g_{exc}^0 w_E \rho _E \tau + g_{inh}^0 w_I \rho _I \tau ) * (\log \dfrac{V_{rest} - V_{st}}{ V_{th} - V_{st} } )^{-1} \end{aligned}$$

**Fig. 1:**
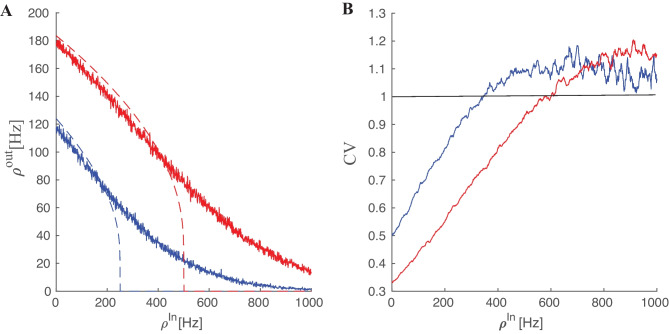
**A** Firing rates of a neuron receiving excitatory Poisson input with two different excitatory rates (the red curve corresponding to the higher one) vs. the Poisson inhibitory input. Dashed lines are the response of the neuron to the constant input with a magnitude equal to the Poisson rates (Eq. 8). **B** Coefficient of variation of the spike intervals of a neuron receiving Poisson inputs of the same rates as in the left graph. Near cutoff, the neuron fires with CV close to one

The left-dashed curves in Fig. 1 show the output firing rate for three different values of excitatory input rate versus inhibitory input rate. In the rest of this section we take the input to the neuron as stationary homogeneous Poissonian inhibitory and excitatory spike trains. In this case the number of spikes in a time interval $\Delta t$ follows a Poisson distribution:

9 $$\begin{aligned} p(k_{[t,t+\Delta T]}) = (\lambda \Delta T)^k \dfrac{e^{ -\lambda \Delta T }}{k!} \end{aligned}$$

The output firing rate of the neuron to the Poisson input is depicted in Fig. 1A. Compared to the constant input with the same constant rate as the Poisson rate $\lambda$, the curve becomes smoother and the transition from silent state to active state does not show a sharp jump. Below the critical inhibition value, the neuron output follows the mean-field deterministic trajectory, however close to this point the fluctuation effect caused by stochastic arrival of spikes manifests itself. Moreover, the stochasticity in the input leads to stochastic firing at the output. Figure 1B shows how the coefficient of variation of the firing time interval of the output spike train change according to the input. This quantity is calculated as

10 $$\begin{aligned} CV(\delta t ) = \dfrac{ \sigma _{\delta t}}{\langle \delta t \rangle } \end{aligned}$$

where $\delta t$ is the set of firing time intervals of the response of the target neuron subjected to a stationary Poisson input. When the excitatory input is much stronger than the inhibitory one the output firing pattern becomes more regular and the CV value is small. However, close to the inhibition cutoff, CV becomes close to unity, which is characteristic of the Poisson point process.

By using diffusion approximation and Fokker-Planck formalism for the time evolution of membrane potential probability density function, we have derived analytic results for average , variance and CV of inter-spike intervals(ISI) of a neuron receiving subthreshhold average input (see SM Sect. 1.2). We have also calculated the gain function of single neurons in the Gaussian approximation and improve the approximation for the potential distribution and the firing rate by considering the auto correlation in the conductance using the $\tau$ expansion method to account for first-order corrections to the Fokker-Planck equation. (see SM Sect. 1.1.1)

We end up with the following expression for the CV of the time interval between spikes:

11 $$\begin{aligned} CV^2 = \dfrac{Var(t)}{\langle t \rangle ^2} \approx 1 + \dfrac{C}{t_1(V_{th} \mid V_{rest})^2} + 2\sqrt{\pi }ln(2) \dfrac{\dfrac{ V_{th} - \langle V \rangle }{b\sqrt{b} \sigma }}{t_1(x_{th} \mid x_0)^2 } \end{aligned}$$

in which,

12 $$\begin{aligned} \begin{aligned} \sigma ^2&= \dfrac{1}{C^2}\{ \tau ^2 g_{exc}^2 w_E^2 \rho _E( v_{Rexc} - \langle V \rangle )^2 + \tau ^2 g_{inh}^2 w_I^2 \rho _I( v_{Rinh} - \langle V \rangle )^2 \} \\ b&= \dfrac{1}{C} (g_{Leak} + g^0_{exc} w_E \rho _E \tau + g^0_{inh} w_I \rho _I \tau ) \end{aligned} \end{aligned}$$

and *C* is a negative constant (see Eq. S22 of SM) that monotonically goes to zero as $x_{th} := V_{th} -\langle V \rangle$ increases. In the limit of large $x_{th}$, the second and the third term both go to zero and *CV* approaches 1. However, in the near threshold approximation the maximum of the third term in Eq. 11 occurs where CV is approaching 1. Expanding in powers of $x_{th}$ , we arrive at

13 $$\begin{aligned} x_{th}^{opt} := V_{th} - \langle V \rangle _{st} = \dfrac{\pi \sigma }{2 \sqrt{b}} \end{aligned}$$

As shown in SM Sect. (1.1.2), $\dfrac{\sigma }{\sqrt{b}}$ reaches a constant value for high input rates. This can be used to determine the value of $\langle V \rangle _{st}$ that leads to maximal CV.

**Fig. 2:**
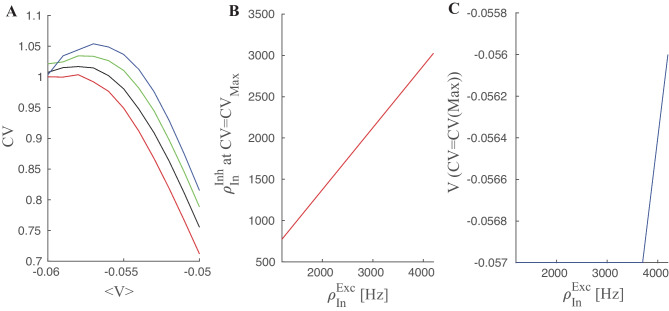
**A** CV of interspike intervals for four different excitatory input rates and their corresponding inhibitory rates, which set the average membrane potential at each specified value shown in the x-axis. (Red curve corresponds to the highest excitatory rate (4000Hz) and the blue one to the lowest rate (1000Hz)) **B** Inhibitory rate vs. excitatory rate at the maximal *CV*. **C** Membrane potential value at the value of the maximal *CV*

Figure 2 shows the CV of the interspike interval for different sets of excitatory and inhibitory pairs of input. As can be seen, at the threshold, neuronal firing time intervals have lower variance, but the CV approaches one far away from the threshold. The stationary membrane potential value corresponding to the maximal value of CV from Eq. 11 is shown in the right diagram and it matches well with the actual values from the simulation. At $V_{P}:= \langle V \rangle _{st}^{opt} \approx -0.56 mv$, the CV for different input rates has a maximum independently of the rate values.

In the middle plot, we see that the inhibitory rate which satisfies $CV=CV_{max}$ varies linearly with the excitatory rates. As can be seen, when the stationary membrane potential is approximately below $V_{P}$, the CV of interspike intervals approaches 1, independently of the values of inhibitory and excitatory rates. This is an indicator that output firing in response to Poisson input is itself a Poisson point process when $\langle V \rangle _{st}$ lies below $V_P$ . For a more conclusive result, one has to calculate higher moments or investigate the limit of the FPT probability density when $x_{th}$ is very large. The fact that the Poisson output condition for different sets of Poisson input leads to approximately a similar level of the membrane potential enables us to introduce the linearization of the output rate at the line corresponding to $\langle V_{m}\rangle = V_p$.

#### Linear Poisson neuron approximation

**Fig. 3:**
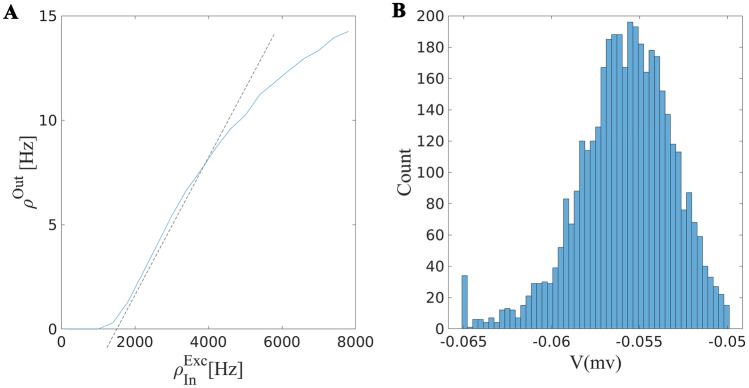
Response of a population of neurons receiving excitatory and inhibitory inputs balanced in a way that the drift term has a fixed point at $V_P = -0.56mv$. **A** Output firing rate for different values of balanced inhibitory and excitatory input rates. The output rate changes semi-linearly on this line and firing in this regime that is driven by the fluctuation in the input causes the neuron to fire with Poisson point process statistics. **B **The stationary potential distribution of the population of neurons. There is a reservoir of neurons close to the threshold while the average firing rate is about 20 Hz. Parameters used: $w_E$ = 0.5, $w_I= 0.75$, $N_E= 7000$, $N_I=0.25* N_E$

Here we want to show that linearizing the response curve of a neuron receiving Poisson current near $V_P$, introduced in the last subsection, leads to a good approximation for the firing rate of the neuron in a wide range of input rates. The linearization is around the line characterized by Eq. 7 with $V_{st}= V_{P}$ in the $\rho _{exc} - \rho _{inh}$ plane. This line corresponds to the balance of mean excitation and inhibition at $V_{P}$. On this balance line, the output rate will depend linearly on the excitatory or inhibitory input rate (see Fig. 3 and E.S21 in SM).

We want to linearize the output rate around $V_P$ . For this purpose let us write the equation of the plane passing through the line of current balance at $V_P$ (Eq. 14) and the tangent line in the $(\rho _E , \rho _{out} )$ plane at some arbitary point $( \rho _{I}^0 , \rho _{E}^0 , \rho _{out}^0)$. The balance condition line for an excitatory neuron connected to $k_{EE}$ excitatory neurons and $k_{IE}$ inhibitory neurons each firing with the rate $\rho _E$ and $\rho _I$, respectively, and receiving external excitatory rate $\rho _{Ext}$ is of the form:

14 $$\begin{aligned} \begin{aligned} \rho _E^e * k_{EE}&= \dfrac{(V_{Rinh} - V_{P})*g_{inh}^0*w_{EI}}{ g_{exc}^0*w_{EE}*(V_{P}- V_{Rexc})} \rho _I *k_{EI} \\&\quad + \dfrac{g_{leak}(V_{rest} - V_{P}) }{\tau * g_{exc}^0*w_{EE}*(V_{P}-V_{Rexc})} - \dfrac{\rho _{Ext}^e}{w_{EE}} \end{aligned} \end{aligned}$$

We rewrite this in simpler form as $\rho _E^e = k \rho _I +C$. The equations for the balance line and the other tangent line in the $(\rho _E , \rho _{out} )$ plane are

15 $$\begin{aligned} \begin{aligned}&\dfrac{ (\rho _E - \rho _E^0) }{k} = \rho _I - \rho _I^0 = \dfrac{ \rho _{out} - \rho _{out}^0 }{\alpha _{OI}} \\&\dfrac{ \rho _O - \rho _O^0 }{\beta _{OE}} = \rho _E - \rho _E^0 \end{aligned} \end{aligned}$$

Therefore, the equation of the plane passing through these lines is of the form

16 $$\begin{aligned} ( \rho _{out} - \rho _{out}^0 ) =&\beta _{OE} ( \rho _E - \rho _E^0) + ( \alpha _{OI}-\beta _{OE}k )(\rho _I - \rho _I^0) \end{aligned}$$

**Fig. 4:**
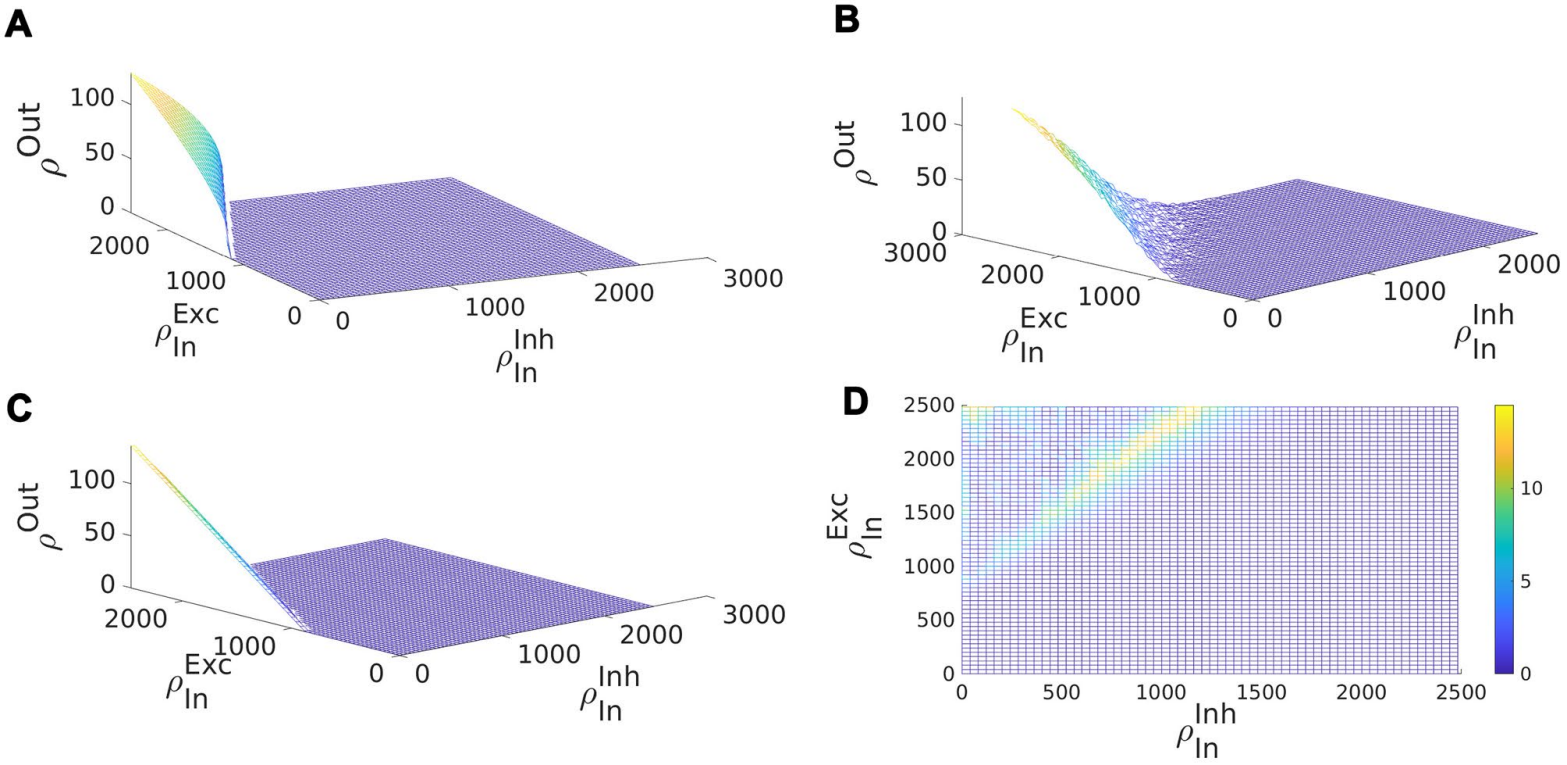
**A** Firing rate of a neuron w.r.t. different values of constant inhibitory and excitatory input. **B** The same for Poisson input. **C** The linear approximation for the output on the critical line of Eq. 14. **D** The error of the linear Poisson neuron approximation

$\beta _{OE}$ is the derivative of the nonlinear response at the selected point in the direction of $\rho _E$, and $\alpha _{OI}$ is proportional to the change of output rate by changing inhibition and accordingly excitation on the balance line. These derivatives do not vary much on the balance line, therefore, the choice of the linearization point does not matter for us at this stage. This suggests that the plane of Eq. 16 is tangent to the $\rho _{out}$ surface. This linear approximation, however, fails for very high excitatory input where the saturation of the neuron causes non-linearity. The linearization point is where the output firing curve has the lowest curvature, and therefore the second derivative vanishes, which makes the approximation error minimal. Figure 4 shows the output firing rate of the target neuron and the linear approximation presented above.

In the next section, we want to investigate the homogeneous firing state of a network. For this purpose we will look at self consistency solutions $\rho _{out} = \rho _E(in) = \rho _E^*$ for an arbitrary value of inhibitory current. From Eq. 16 :

17 $$\begin{aligned} (1 - \beta _{OE}) \rho _E^* = ( \alpha _{OI}-\beta _{OE}k )(\rho _I - \rho _I^0) + \rho _O^0 - \beta _{OE}\rho _E^0 \end{aligned}$$

Putting in $k\rho _I^0 - \rho _E^0 = -C$ and dividing the above equation by $\beta _{OE}$, we arrive at

18 $$\begin{aligned} (\dfrac{1}{\beta _{OE}} - 1 ) \rho _E^* = - k\rho _I -C + \dfrac{\alpha _{OI}}{\beta _{OE}} (\rho _I - \rho _I^0 ) + \dfrac{1}{\beta _{OE}}\rho _O^0 \end{aligned}$$

$\beta _{OE}$ depends on the number of excitatory input to the cell, $K_{EE}$, and is related to the proportional change of output firing at the balance line to the change in the firing rate in each excitatory neuron. On the other hand, $\alpha _{OI}$, proportional to change in the firing rate while fixing the balance condition, is much smaller than $\beta _{OE}$. Therefore, when $K_{EE}$ is large, the self-consistency equation matches the balance line of Eq. 14 with a minimal error.

### Sparse homogeneous EI population dynamics

As we have seen for sets of Poisson input that produce low firing output the statistics of spiking events resembles a Poisson process. In a population of neurons there might be a stable stationary or oscillatory population rate with Poisson firing of individual neurons. In this case, the magnitude of fluctuations in the population average scales as *O*(*N*). This inhomogeneous synchronous or asynchronous firing state exists only in the low firing regime. In the high firing state, the large imbalance of excitatory and inhibitory input leads to periodic firing of the individual neurons which can be also synchronized with high amplitude and high frequency oscillatory population rates. We can use the linear Poisson approximation for identifying and analyzing the dynamics in the low firing rate regime which is of most interest to us. In a homogenous population, solutions of the self-consistency equations for both inhibitory and excitatory neurons’ average output firing rate receiving synaptic currents originated from both neurons in the population and external inhibitory and excitatory currents $\lambda$ can be written as follows:

19 $$\begin{aligned} \rho _{E}^{st}&= f( k_{EE}\rho _{E}^{st} , k_{EI} \rho _{I}^{st} ,\lambda _{EE} , \lambda _{EI} ) \end{aligned}$$

20 $$\begin{aligned} \rho _{I}^{st}&= g( k_{EI}\rho _{E}^{st} , k_{II} \rho _{I}^{st} ,\lambda _{IE} , \lambda _{II} ) \end{aligned}$$

for $\rho _{E} , \rho _{I} \in [0, \rho _{max} ]$ . Functions *f* and *g* are called excitatory and inhibitory gain functions and $k_{xy}$ is the number of internal connections between neurons in the population. Solving for these gain functions in the general case is not analytically tractable for the EI population. Dynamics to the stationary rates given by Eqs. 19-20 can be phenomenologically approximated by the following mean field equations:

21 $$\begin{aligned} \begin{aligned} \dfrac{d\rho _{E}}{dt}&= - \dfrac{1}{\tau _m}(\rho _E(t) - f( k_{EE}\rho _{E}(t) , k_{EI} \rho _{I}(t) ,\lambda _{EE} , \lambda _{EI} )) \\ \dfrac{d\rho _{I}}{dt}&= -\dfrac{1}{\tau _m}(\rho _I(t) - g( k_{EI}\rho _{E}(t) , k_{II} \rho _{I}(t) ,\lambda _{IE} , \lambda _{II} ) ) \end{aligned} \end{aligned}$$

This set of equations may have multiple solutions and changing control parameters can lead to Hopf and saddle-node bifurcations, which in turn produce/destroy oscillations or produce/destroy pairs of fixed points. Although it is possible to numerically investigate the Fokker-Planck equations (FPE) for probability density functions of membrane potentials in the EI population and its bifurcation diagram, in the next subsections, we follow another approach by using linearized nullclines approximation and logistic function approximation for functions *f* and *g*. We show that studying these model systems is appropriate for the bifurcation analysis and agrees with simulation results.

#### Linearized nullclines

**Fig. 5:**
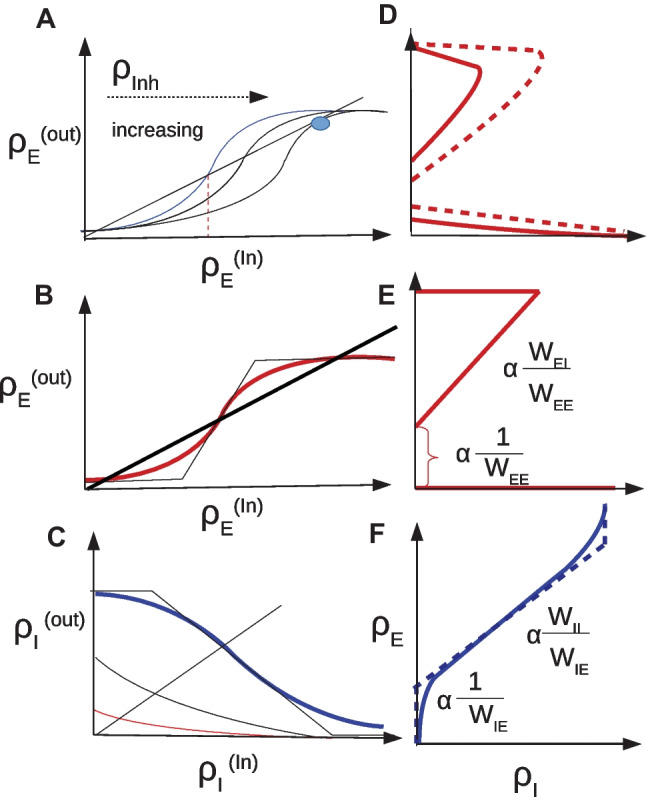
**A** Excitatory neuron output rate vs. excitatory input rate at three fixed values of inhibitory currents. Increasing Inh. current shifts the gain function to the right. The intersection of the curve and the line $\rho _E^{out} = \rho _E^{In}$ are fixed points of the firing rate equation in the homogenous network. **B** Linearized excitatory gain function. Linearization is performed at the inflection point of the curve and the output firing rate is bounded to the interval $[0,\rho _{max}]$ **C** Inhibitory neuron output rate vs. inhibitory input at three different values of excitatory current.Increasing Exc. current shifts the gain function to the right. The intersection of the curve and the line $\rho _I^{out} = \rho _I^{In}$ are fixed points of the firing rate equation in the homogenous network. **D** Excitatory nullclines of Eqs. 19-20 for two different values of $w_{EE}$ with the dotted curve corresponding to the higher value. **E** Linearization of the excitatory nullcline **F** Inhibitory nullcline and its linearization based on Eqs. 22-23 (dotted curve)

Function *f* in the Eq. 19 for the stationary excitatory rate is of the form of an S-shape or sigmoidal curve. Therefore, this equation has one or three solutions depending on the value of the inhibitory rate. This is shown in Fig. 5A for three different total inhibitory currents. Figure 5D shows the solutions to the Eq. 19 for a typical sigmoidal gain function and different values of total inhibitory current to the excitatory population. This is plotted for two different values of $w_{EE}$ with the dashed curve corresponding to higher $w_{EE}$.

Similarly, Fig. 5C is the plot corresponding to the Eq. 20. Here, the nonlinear sigmoid function *g* is plotted for three different values of excitatory current. There exists a single intersection between the line passing through the origin and these curves, which means Eq. 20 has a unique solution for the stationary inhibitory rate at each specific excitatory input. Figure 5F is the plot of the location of these intersections for different values of inhibitory input. As can be seen in Fig. 5, there exists a semilinear section in the nullcline graphs corresponding to solutions in the linear Poisson section of gain functions. Based on the linear Poisson approximation of the Sect. 3.1.1, the equations for these lines in both excitatory and inhibitory nullcline graphs are:

22 $$\begin{aligned} \begin{aligned} \rho _E^{exc} * k_{EE}&= \dfrac{(V_{Rinh} - V_{P})*g_{inh}^0*w_{EI}}{ g_{exc}^0*w_{EE}*V_{P}} \rho _I *k_{EI} \\&\quad + \dfrac{g_{leak}(V_{rest} - V_{P}) }{\tau * g_{exc}^0*w_{EE}*V_{P}} - \dfrac{\lambda _{EE}}{w_{EE}} \end{aligned} \end{aligned}$$

23 $$\begin{aligned} \begin{aligned} \rho _E^{inh} * k_{IE}&= \dfrac{(V_{Rinh} - V_{P})*g_{inh}^0*w_{II}}{ g_{exc}^0*w_{IE}*V_{P}} \rho _I * k_{II} \\&\quad + \dfrac{g_{leak}(V_{rest} - V_{P}) }{\tau *g_{exc}^0*w_{IE}*V_{P}} - \dfrac{\lambda _{IE}}{w_{IE}} \end{aligned} \end{aligned}$$

where $k_{\alpha \beta }$ is the number of excitatory/inhibitory synapse to an excitatory/inhibitory neuron. In the remainder of this work, we assume external inhibiotry currents to be zero, in line with our assumption that inhibition is local in our model. We assume $\dfrac{k_{EI}}{k_{EE}} = \dfrac{k_{II}}{k_{IE}}$, which simplifies our analysis.

In the $\rho _{I} - \rho _{E}$ plane the slope and the *y*-intercept of the two lines in Eqs. 22-23 determine the intersection of the two nonlinear nullclines and can be used to find approximate locations of the bifurcation points of Eq. 21. We choose $\langle w_{EE}\rangle$ and $\rho _{Ext}=\lambda _{EE}$ as control parameters of our model. Therefore, we first discuss how their change affects the nullclines of Eqs. 19-20. Increasing $\rho _{Ext}$ moves the sigmoid graph in Fig. 5A upwards causing the low and middle fixed points to move towards each other. For a sufficiently high value of excitatory rate, these fixed points will disappear by a saddle-node bifurcation. In the excitatory nullcline graph (Fig. 5D) increasing $\rho _{Ext}$ shifts the graph to the right. Increasing $W_{EE}$ will both reduce the *y*-intercept of the excitatory nullcline and the slope of the linear section as shown in Fig. 5D. The nullcline for the inhibitory rate equation stays intact under change of control parameters.

The intersections of the inhibitory and excitatory nullclines are solutions of the set of rate Eqs. 19-20. Based on the number of fixed points and their stability, the system can show bi-stability of quiescent and high firing, oscillatory dynamics, avalanches, high synchronized activity, and quiescent state. Investigating the linearized sections of the graphs can help us identify different regimes of activity. The slope and *y*-intercept of the linear sections of both nullclines can be compared for this purpose. Based on the Poisson neuron approximation there exists a point in control parameter space where the *y*-intercept and slope of two nullclines are equal. This point is the solution of the following linear constraints:

24 $$\begin{aligned} s_{exc} := \dfrac{ w_{EI}k_{EI}}{w_{EE}k_{EE}} = \dfrac{ w_{II}k_{II}}{w_{IE}k_{IE}} := s_{inh} \end{aligned}$$

25 $$\begin{aligned} y_{exc} := \dfrac{ d - \rho _{Ext}}{w_{EE}k_{EE}} = \dfrac{ d - \lambda _{IE}}{w_{IE}k_{IE}} := y_{inh} \end{aligned}$$

where *d* is a constant equal to $\dfrac{g_{leak}(V_{rest} - V_{P}) }{\tau * g_{exc}^0*(V_{P}-V_{Rexc})}$.

**Fig. 6:**
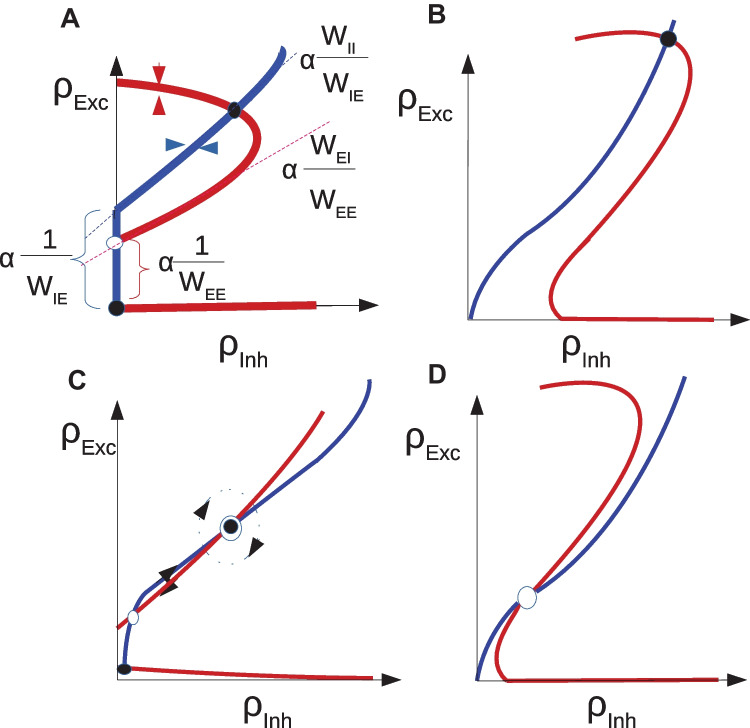
Nullcline diagrams corresponding to regimes of bistability (**A**), high synchronized firing (**B**), avalanches (**C**), and oscillatory dynamics (**D**). Red curves are excitatory nullclines (Eq. 19) and blue curves are inhibitory nullclines (Eq. 20). Filled and empty circles represent stable and unstable fixed points, respectively. In (A), the linearized nullclines obtained from linear Poisson approximation are drawn and the dependence of their slope and y-intercept on weights is shown. (Eqs. 22-23). In (**C**), the fixed point on the semilinear section is weakly stable or unstable based on the sign of the eigenvalue of the Jacobian with the absolute value near zero

Figure 6A shows the case in which $w_{EE}w_{II} > w_{EI}w_{IE}$ and the *y*-intercept of the excitatory nullcline is lower than the inhibitory one. This occurs in the regime of a low to moderate imbalance of excitatory and inhibitory external input and high excitatory synaptic weight. In this case, the quiescent and the high firing fixed point are both stable and separated by a saddle. Increasing external excitatory input, the excitatory nullcline are shifted to the right and the middle saddle and quiescent node disappear by a saddle-node bifurcation and only the high firing synchronous state remains (Fig. 6B). Increasing $w_{EE}$ has the same qualitative effect. However, decreasing external input or $w_{EE}$ drives the system to a quiescent state through different sets of bifurcations depending on the initial state of the system and in general on other parameters of the model. This intermediate transition state involves the appearance of a fixed point in the linear section.

When $s_{exc} > s_{inh}$ while $y_{exc} < y_{inh}$, there is a fixed point in the linear section as depicted in Fig. 6C. We will discuss the stability of the fixed point on the linear segment in the following sections. By increasing external input, the quiescent fixed point and the low saddle move closer to each other while the fixed point on the linear section ascends to higher rate values. After the saddle-node bifurcation at the low rate, only the fixed point on the linear section survives as shown in Fig. 6D. These two arrangements when the fixed points are close to low firing regimes are important for us because of the avalanche dynamics that appear near this region. The intersection point of the nullclines in the semilinear regime can be approximated by the intersection point of the linearized nullclines which is:

26 $$\begin{aligned} \begin{aligned} \rho _E^c&= \dfrac{\tau g_0 (V^R_{E}-V_{P})(c_{II}\rho _{Ext} - c_{EI}\lambda _{IE}) + g_L(V_L - V_{P})(c_{II}-c_{EI})}{\tau ( c_{IE}c_{EI}-c_{EE}c_{II})} \\ \rho _I^c&= \dfrac{\tau g_0(V^R_{E}-V_{P})(c_{EE}\lambda _{IE}-c_{IE}\rho _{Ext}) - g_L(V_L - V_{P})(c_{IE}-c_{EE})}{\tau (c_{IE}c_{EI}-c_{EE}c_{II})} \end{aligned} \end{aligned}$$

where $$c_{xy} = k_{xy} w_{xy} g_{y}(V^R_{y}-V_{P})$$ .

**Fig. 7:**
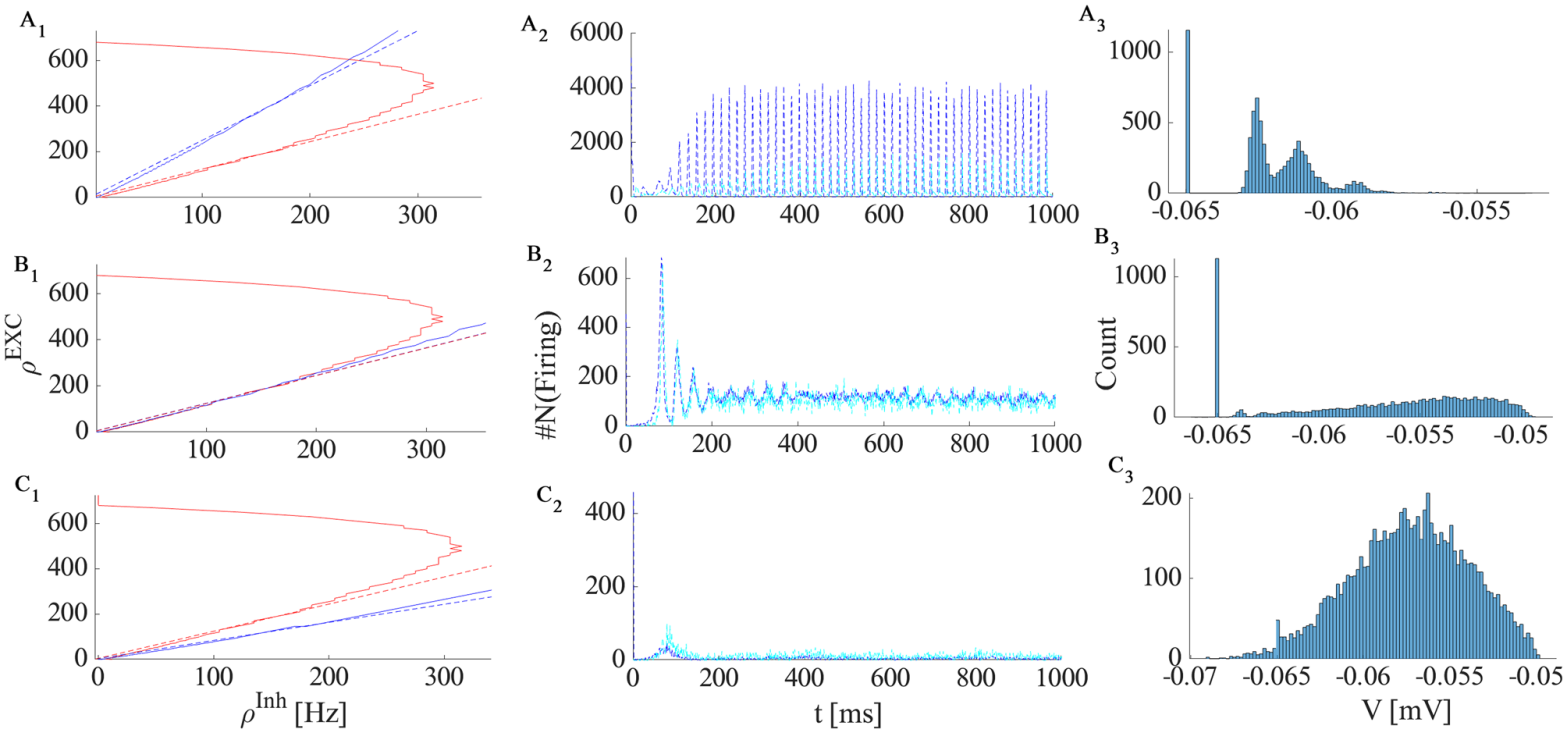
**A1**-**B1**-**C1** Nullclines for excitatory and inhibitory neuron populations and their corresponding linear approximations of Eqs. 22-23 obtained from network simulation. Values of parameters are $w_{EE}= [0.75$ (**A**), 0.55 (**B**), 0.4 (**C**)]$, w_{EI}= 2 , w_{II} =1.5 , w_{IE} = 0.6$. **A2**-**B2**-**C2** Number of active excitatory neurons (dark blue) and active inhibitory neurons (light blue) in each time slot of (0.1ms) for three different values of $w_{EE}$. **A3**-**B3**-**C2** The corresponding stationary membrane potential distribution. In the asynchronous state, the distribution has higher variance

As discussed previously, in the intermediate parameter range, the high fixed point might become unstable through either an Andronov-Hopf or a saddle node bifurcation. Figure 7 shows nullcline graphs and population activity when the high fixed point loses stability by a Hopf bifurcation. Figure 7A shows nullclines population activity of a system that has stable high and quiescent fixed points with a saddle node at low rates. By decreasing $w_{EE}$, $s_{exc}$ approaches $s_{inh}$, while sufficient external input guarantees that $y_{exc} <y_{inh}$ during this parameter change. In this particular setup, the inhibitory nullcline is semi-linear and we may speculate that the high fixed point goes through a Hopf bifurcation when the return point of the excitatory nullcline touches the inhibitory nullcline which takes place at some value $w^*_{EE} \in [0.55,0.75]$. Decreasing $w_{EE}$ further, the high saddle node descends through a linear segment and gets closer to the lower saddle point (Fig. 7B). The limit cycle becomes unstable by a saddle separatrix loop bifurcation. After saddle-node annihilation of low and high saddles,the system will end up in the quiescent state for low values of $w_{EE}$ (Fig. 7C). Neurons are firing synchronously at a high rates in three different sub-populations in the first case. The high oscillatory activity appears in the second regime where the unstable saddle, which is encircled by a stable limit cycle, lies close to the high activity region. The membrane potential distribution, in this case, has a higher variance, and neurons fire asynchronously.

**Fig. 8:**
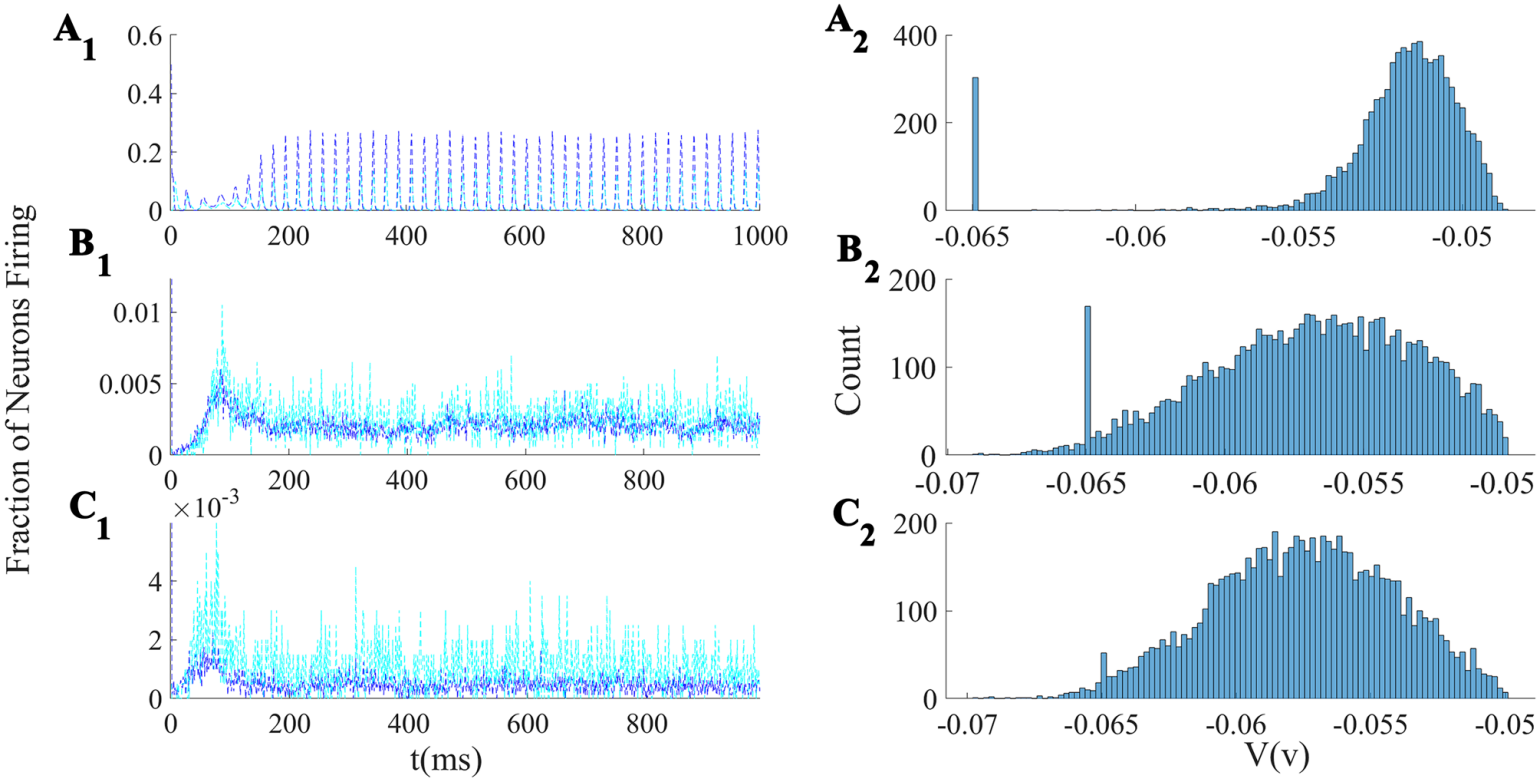
Simulation results of the network with same parameters as in Fig. 7 except for $w_{II} =2.4$. The EI population shows asynchronous firing in the medium range of $w_{EE}$. This suggest that there is a stable fixed point at the intersection of the linear segments of the excitatory and inhibitory nullclines

In addition to oscillatory activity in the middle range of rates, the EI-population can exhibit non-oscillating asynchronous activity which corresponds to a stable fixed point in the linear regime. Figure 8 is the simulation result of the population rates similar to the setup of the Fig. 7 with higher $W_{II}$, which, as we will see later, makes the fixed point on the linear section stable.

#### Logistic function approximation of gain functions

In this section, we approximate gain functions by logistic functions to analyze bifurcation diagrams and approximate locations of bifurcaion points. For this purpose, we consider the gain functions in the following form:

27 $$\begin{aligned} \begin{aligned} g^x(\rho _{Inh} , y_x)&= \dfrac{\rho _{max}}{1 + \alpha (\rho _{Inh})e^{-ky_x}} - z_0, \\ y_x&= g_{syn}\tau w_{xI} \rho _{Inh} ( V_{Rinh} - V_{th}) + g_{syn}\tau (w_{xE}\rho _{exc} + \rho _{Ext}^x) (V_{Rexc}-V_{th}) \\&\quad +g_{L}(V_{Le ak}-V_{th}), \\ z_0&= \dfrac{\rho _{max}}{1 + \alpha (0)e^{-kg_{L}(V_{Le ak}-V_{th})}} \end{aligned} \end{aligned}$$

Here, *x* stands for either excitatory (E) or inhibitory (I) gain functions, which have the same form but different input arguments. $\rho _{Ext}^x$ is the external excitatory input to the population *x*.

**Fig. 9:**
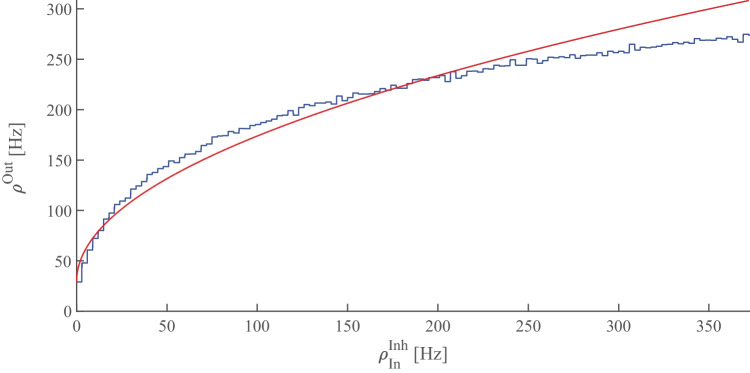
Output rate as a function of input inhibitory rate (blue curve), when the excitatory rate is selected in a way that average membrane potential of the neuron is $V_{th}$. Neuron is operating near a saddle-node bifurcation point at which $F(I_{syn}) = k\sqrt{I_{syn}-I^*}$ (red curve)

At $y_x=0$, balanced input sets the membrane potential at the threshold value and the output rate is approximately $g_{th} = \dfrac{\rho _{max}}{1+\alpha (\rho _{Inh})}$. Dependence of the output rate on inhibitory input, when the balance condition at threshold holds, is represented by the function $\alpha$. At $y=0$, the output rate is proportional to the standard deviation in the input and it can be written as function of the inhibitory input rate as (see Fig. 9):

28 $$\begin{aligned} g_{th} = b_0 + b_1 \sqrt{\rho _{Inh}} \end{aligned}$$

which fixes the function $\alpha (\rho _{Inh})$.

At equilibrium, the population rates satisfy:

29 $$\begin{aligned} \begin{aligned} \rho _{I}&= g^I(\rho _{I} , c_{IE}\rho _{E} + c_{II}\rho _{I} + d\rho _{Ext}^I) - z_0 \\ \rho _{E}&= g^E(\rho _{I} , c_{EE}\rho _{E} + c_{EI}\rho _{I} + d\rho _{Ext}^E) -z_0 \end{aligned} \end{aligned}$$

where $c_{xy} = c k_{xy} w_{xy}(V_{R_y} - V_{th})$. As before, we take $w_{EE}$ and $\rho _{Ext}^E$ as control parameters. Therefore, the solution of the first equation in Eq. 29 is independent of control parameters and gives a curve in the $\rho _{I}-\rho _{E}$ plane. Taking into account that the inverse of $g(\rho _{Inh} , y)$ is $g^{-1}(\rho _{Inh} , z) =\dfrac{1}{k}( \log (\dfrac{z}{\rho _{max} -z}) + \log (\alpha ))$, the equation for the inhibitory nullcline can be written as :

30 $$\begin{aligned} \rho _{E} =&\dfrac{1}{c_{IE}}(\dfrac{1}{k}[\log (\dfrac{(\rho _{I}+z_0)}{\rho _{max} -(\rho _{I}+z_0)}) + \log (\alpha )] - c_{II}\rho _{I} - d\rho _{Ext}^E - g_L ) \end{aligned}$$

The term in brackets accounts for non-linearity in low and high values of $\rho _{I}$. The derivative of this term w.r.t. $\rho _I$ is $\dfrac{\rho _{max}}{\rho _{I}(\rho _{max} -\rho _{I})}$, which is very small in the middle range of $\rho _{I}$ at values close to $0.5 \rho _{max}$. This is consistent with the fact that nullclines are approximately linear in the middle range of the rates.

To analyze linear stability of the fixed points, we compute derivatives of the gain function:

31 $$\begin{aligned} \begin{aligned} \dfrac{\partial g^x}{\partial \rho _E}&= kc_{xE} g^x(1-\dfrac{g^x}{\rho _{max}}) \\ \dfrac{\partial g^x}{\partial \rho _I}&= kc_{xI} g^x(1-\dfrac{g^x}{\rho _{max}}) - \dfrac{1}{\alpha } g^x(1-\dfrac{g^x}{\rho _{max}})\dfrac{\partial \alpha }{\partial \rho _I} \end{aligned} \end{aligned}$$

Here $g^x$ stands for $g^I$ or $g^E$. One can substitute $\rho _I + z_0$ and $\rho _E + z_0$ from Eq. 29 for $g^I$ and $g^E$, respectively. Therefore, the Jacobian matrix components at the fixed point are:

32 $$\begin{aligned} \begin{aligned} J_{11}&= -1+c_{EE}\rho ^E(1-\dfrac{\rho _E}{\rho _{max}}) \\ J_{12}&= c_{EI}\rho ^E(1-\dfrac{\rho ^E}{\rho _{max}}) - \dfrac{1}{\alpha }\rho ^E(1-\dfrac{\rho ^E}{\rho _{max}})\dfrac{\partial \alpha }{\partial \rho ^I} \\ J_{21}&= c_{IE}\rho ^I(1-\dfrac{\rho ^I}{\rho _{max}}) \\ J_{22}&=-1+ c_{II}\rho ^I(1-\dfrac{\rho ^I}{\rho _{max}}) - \dfrac{1}{\alpha }\rho ^I(1-\dfrac{\rho ^I}{\rho _{max}})\dfrac{\partial \alpha }{\partial \rho ^I} \end{aligned} \end{aligned}$$

Hopf bifurcation occurs at fixed point solutions at which the trace of the Jacobian vanishes and its determinant is positive. On the other hand, at saddle-node bifurcation occurs at points where the determinant vanishes. We proceed to approximate local bifurcation lines in the parameter space.

The condition on zero trace $Tr(J) = J_{11}+J_{22}=0$ parameterized by the inhibitory nullcline curve (Eq. 30) determines the value for $w_{EE}$ at which a Hopf bifurcation can occur. Next, we should check the positivity of the determinant to sketch the Hopf bifurcation line in the $w_{EE}-\rho _{Ext}^E$ plane. A point where both determinant and trace of *J* are zero, is called a *Bogdanov-Takens* (BT) *bifurcation point*. In Sect. (2.1) of SM, we showed that when $\mid c_{II}c\rho _I (1 - \dfrac{\rho _I}{\rho _{max}}) \mid$ is at a moderate value, i.e. sufficiently greater than one, at the BT point we have:

33 $$\begin{aligned} c_{EE}^{BT} \approx \dfrac{c_{IE}c_{EI}}{c_{II}} \end{aligned}$$

If we take number of connections to satisfy $\dfrac{k_{EI}}{k_{EE}} = \dfrac{k_{II}}{k_{IE}}$, then $w_{EE}^{BT} = \dfrac{w_{IE}w_{EI}}{w_{II}}$. To sketch the saddle-node bifurcation line we should look at solutions to $det(J) = 0$. Inserting $\rho _E(\rho _I)$ from Eq. 30 into *det*(*J*) , for each point on the inhibitory nullcline, there exists some $w_{EE}$ for which $det(J)=0$. The only condition to check is $w_{EE}>0$. Again the condition that the excitatory nullcline intersects the inhibitory one at the fixed point determines $\rho _{Ext}$. Along the semi-linear section of the nullcline, the condition $det(J)=0$ translates into the alignment of the slopes of the linearized nullclines. Therefore, along this section $w_{EE}$ varies very little.

**Fig. 10:**
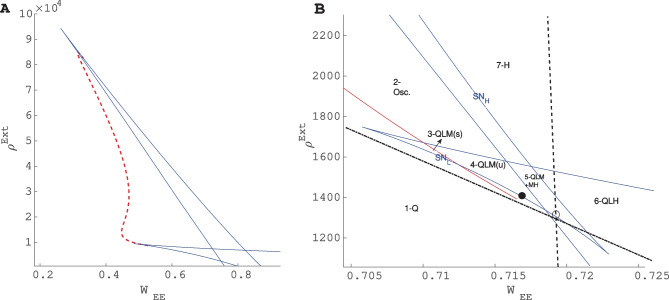
**A** Local bifurcation diagram in the control parameter plane $(W_{EE} , \rho _{Ext})$. The red curve is the Hopf bifurcation line and the blue curves are saddle-node bifurcation lines. The free parameters of the model are $\rho _{Ext}^{Inh} =300Hz,W_{II}=1,W_{EI}=1.8$ and $W_{IE}=0.6$. **B** Zoom in on the local bifurcation diagram at low firing rates and the corresponding regimes of phase space with different numbers of fixed points. The dashed line is the condition on the equal slope of linearized nullclines and the semi-dashed the line is the condition on equal *y*-intercepts. The BT point (black dot) is close to the intersection of these lines. In the labeling of regions (Q) denotes the quiescent state fixed point, (L) is the fixed point at low firing rate, (M) is the fixed point in the linear section, and (H) is the high firing fixed point. Region (1) corresponds to the system with the stable quiescent fixed point. In region (2), the only fixed point is unstable and the firing pattern is oscillatory. In region (3), the fixed point in the linear section is stable while in the region (4) it is unstable. Increasing $W_{EE}$, by saddle-node bifurcation in the high firing rate branch, another pair of stable and unstable fixed points emerges (Region 5). Region (6) corresponds to the system with bistability of high firing and quiescent states. In region (7), only a high firing fixed point exists

Figure 10A shows Hopf and saddle-Node bifurcation lines with parameters written in the caption. As can be seen, there exist two Bogdanov -Takens bifurcation points at low and high values of external input corresponding to the intersection of nullclines in low and high firing rate regimes.

Figure 10B is the bifurcation diagram at low rates. Different regimes of phase space corresponding to different numbers and/or types of fixed points have been labeled. The system has between one and five fixed points. Region (1) with low values of $W_{EE}$ and external input strength is the quiescent state with only one stable fixed point. In region (2), there is only an unstable fixed point surrounded by a stable limit cycle corresponding to the intersection of nullclines in the semi-linear sections. In regions (3) and (4) near the BT point, two other fixed points exist at low firing rates. The type of solution in these regions will be discussed later in this section. Region (5) corresponds to the case where there exist 5 intersection points on the nullcline map and the bi-stability of the quiescent and the high state which survives after the annihilation of unstable nodes on the middle section of the nullcline to the region (6). Finally, in the region (7), at high external input and synaptic weight, the only existing fixed point is the high firing one.

Dashed lines are the constraints of Eqs. 24-25 corresponding to equal slope and *y*-intercept of the linearized nullclines. The vertical line is the value of $w_{EE}^*$ that matches the slopes, for $w_{EE}< w_{EE}^*$ the inhibitory feedback is getting stronger. The oblique line shows values of $\rho _{Ext}$ for each $w_{EE}$ that equalize *y*-intercepts of linearized nullclines. In the region below this line $y_{inh}< y_{exc}$ and vice versa.

#### Dynamics near the BT bifurcation point

**Fig. 11:**
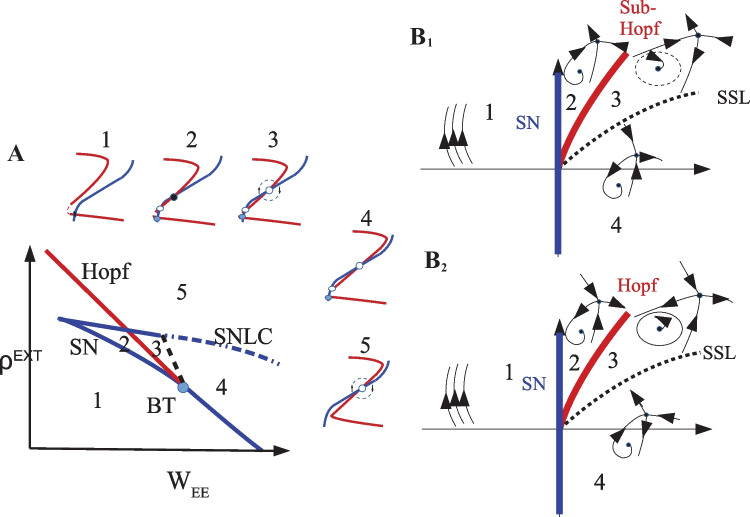
**A** Exc. (red) and Inh.(blue) nullclines near the BT point corresponding to the system in each state shown in the bifurcation diagram in which the black dashed line is the saddle-node separatrix-loop bifurcation and the blue dotted-dashed is the saddle-node on limit cycle (SNLC) bifurcation line. Filled and empty circles represent stable and unstable fixed points, respectively. (**B**$_1$) Dynamic flow near the high BT point and (**B**$_2$) the low BT point. Blue lines are saddle-node bifurcations, red lines are Hopf bifurcations, and dashed lines are saddle-node separatrix loop bifurcations

The exact locations of the *BT* points $(c_{EE}^{BT},\rho _{Ext}^{BT} , \rho _{E}^{BT},\rho _I^{BT})$ are solutions of $det(J) =Tr(J) =0$ and $g_E(i_E)=g_I(i_I) = 0$. Figure 11A shows nullcline arrangements near the low BT point and the global saddle separatrix loop bifurcation line which annihilates the limit cycle solution of the region (3), shown in the same figure.

In the previous section, we showed that the low BT point is located close to the matching condition for the *y*-intercept and the slopes of the linearized nullclines, which we rewrite here:

34 $$\begin{aligned} \begin{aligned} c_{EE}^*&= \dfrac{c_{IE}c_{EI}}{c_{II}} \\ \rho _{Ext}^{E^*}&= \dfrac{c_{EE}^*}{c_{IE}}(\rho _{Ext}^I-d) +d \end{aligned} \end{aligned}$$

where *d* is a constant defined in Eq. 25 .

At the BT point the linearized matrix is of the form:

35 $$\begin{aligned} J_{BT} = \begin{pmatrix} \alpha &{} -\beta \\ \dfrac{\alpha ^2}{\beta } &{} -\alpha \end{pmatrix} \end{aligned}$$

where $\dfrac{\beta }{\alpha } = \dfrac{c_{EI}}{c_{EE}} = \dfrac{c_{II}}{c_{IE}}$. At the BT point, the Jacobian has a double zero eigenvalue and with proper coordinate transformation, it can be written in the form:

$$\begin{aligned} J = \begin{pmatrix} 0&{} 1\\ 0 &{} 0 \end{pmatrix} \end{aligned}$$

As shown in Fig. 11, near the BT point apart from local bifurcations, i.e., Hopf and saddle-node, there is saddle-node separatrix loop bifurcation which annihilates the stable or unstable limit cycle that is produced by a super- or sub-critical Hopf bifurcation, respectively. By writing the normal form for the BT bifurcation, we can analyze the type of dynamic flow in the vicinity of low BT point (see SM Sect. 2).

However, linearization near the BT point can help us to identify regimes surrounding it without having to calculate the normal form parameters. Nullcline maps related to regions (2) and (3) in Fig. 11A shed light on the type of BT bifurcation. In the plot corresponding region (3), $w_{EE}$ is higher which means that the Jacobian at the fixed point has lower determinant and higher trace. Of the two fixed points in regions (2) and (3) at the semi-linear section the one in the higher $W_{EE}$ regime is the unstable point. Therefore, in our case near the low BT point the phase space resembles the one in Fig. 11B$_2$. Increasing $w_{EE}$ from region (2) will result in loss of stability of the fixed point in the linear branch by Hopf bifurcation, as the trace of the Jacobian at the fixed point becomes zero. However, as we increase the $w_{EE}$, slope of the linearized approximation of the nullclines which are tangent to the stable and unstable manifolds of the saddle point that separate the quiescent fixed point and the limit cycle solution, get closer to each other. At some point, these manifolds cross over and therefore destroy the limit cycle solution through a saddle-node separatrix loop bifurcation and we end up with a fixed point of source type at the intersection of nullclines in the linear firing regime of region (4) in Fig. 11A.

#### Avalanches in the region close to the BT point

We assume that the external input to both excitatory and inhibitory neurons is dominated by the excitatory type and that connections among excitatory populations have a longer range. Therefore, the external excitatory input to the excitatory population is higher than to the inhibitory one. On the other hand, inhibitory connections are local and therefore, follow the dynamics of the adjacent excitatory population. Strong local feedback provided by inhibition prevents the excitatory network to be overloaded. However, it is very closely balanced to set the network near the threshold of activation so that the system can respond efficiently to external input. In the background regime of spontaneous activity, the EI population shows avalanche pattern dynamics and oscillatory behavior. Synchronization of oscillations and the scale-free avalanche dynamics are characteristic behaviors experimentally validated Beggs and Plenz (2003); Gong et al. (2003); Meisel et al. (2013) . In the sequel, we will see that close to the BT at a low firing rate regime, we can observe both phenomena.

In the parameter space enclosed by Hopf and saddle-node bifurcation lines, i.e., region (4-QLM(u)) in Fig. 10B, there exist regions with both oscillatory and medium-range Poisson firing states. Decreasing $W_{EE}$ while changing $\rho _{Ext}^E$ accordingly, so that the low and medium fixed points move closer to the origin, the system moves towards the Bogdanov-Takens bifurcation point, where the saddle-node bifurcation and Hopf bifurcation lines intersect. In this regime, we see avalanche dynamics in our population. Close to the BT point, the basin of attraction of the quiescent fixed point shrinks and the noise level is high enough for escaping from it. This is in the adjacency of both the saddle-node bifurcation, which creates an unstable low and a weekly stable medium firing fixed point, and the Hopf bifurcation of the quiescent fixed point. This region corresponds to strong inhibitory feedback and sufficient imbalance in external excitatory input. In the nullcline graph, this translates into the state where the *y*-intercept of the excitatory graph is slightly lower than the *y*-intercept of the inhibitory graph and the slope of the excitatory is larger than the slope of the inhibitory one. Increasing $W_{EE}$ causes the middle fixed point to move to higher rates and to have a larger basin of attraction. On the other hand, the saddle and the quiescent fixed point move towards each other in the phase diagram and annihilate each other at the saddle-node bifurcation.

**Fig. 12:**
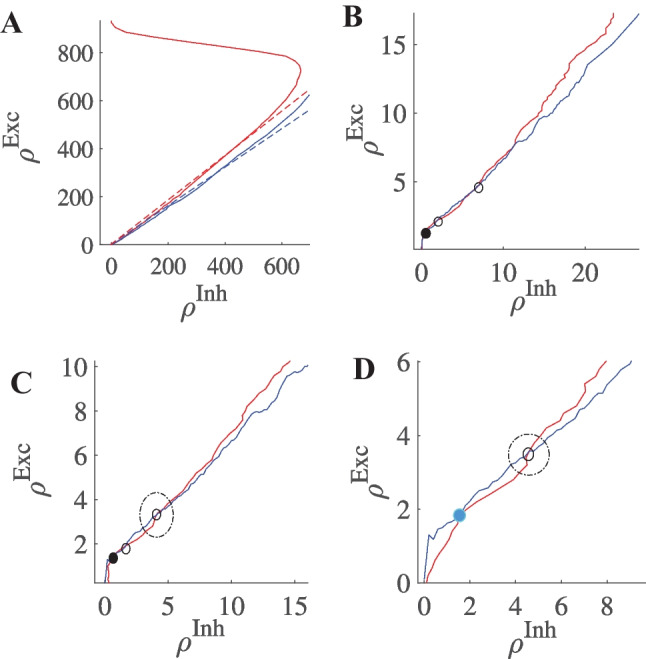
Nullcline configuration around the avalanche dynamic region. Red curves are excitatory nullclines and blue curves are inhibitory nullclines. Filled and empty circles represent stable and unstable fixed points, respectively. **A** Slopes of linearized nullclines match close to the BT point. **B** Near the BT point, the basin of attraction of the quiescent fixed point has a low volume, and saddle-node bifurcation produces pair of fixed points in the low firing rate regime, here both are unstable. **C** In a specific parameter regime, a limit cycle solution exists in the vicinity of the unstable fixed point. **D** In higher external input quiescent state vanishes and the low rate limit cycle remains leading to quasi-periodic rates

Figure 12 shows nullcline arrangements in the region where we observe avalanche patterns. Figure 12A is the general position of nullclines indicating the fixed point in the linear regime. The other three diagrams correspond to two regimes near the BT point and transition between these two. The diagram in Fig. 12B belongs to the section to the right of BT where there exists a quiescent fixed point with a weakly unstable saddle in the linear section. Here noise causes the system to escape from the basin of attraction of the fixed point which then relaxes in the direction of the nullclines. As nullclines lie on top of each other, the decay time is large and the system shows high synchronous activity while returning to a quiescent state. An increase of external drive or decrease of $W_{EE}$ leads to saddle-node annihilation which leaves the system with a fixed point at the middle section. Figure 12C belongs to the state on the left side of BT1 in the vicinity of Hopf bifurcation of the origin. In this case, there is a limit cycle around the saddle point in the linear branch. Like the previous case, adjacency of the fixed point at the origin to the saddle shrinks the basin of attraction of the quiescent state, and therefore noise can bring the system to the limit cycle which itself is sensitive to internal and external noise. Finally, Fig. 12D shows how saddle-nodes of the last two diagrams are annihilated by saddle-node on limit cycle and saddle-node bifurcations, respectively. Here a limit cycle solution emerges. However, close to the origin this limit cycle stays for a longer time in the lower section of very low firing because of slow flow in this region. The outcome is again a quasi-periodic burst of avalanches followed by a quiescent state.

**Fig. 13:**
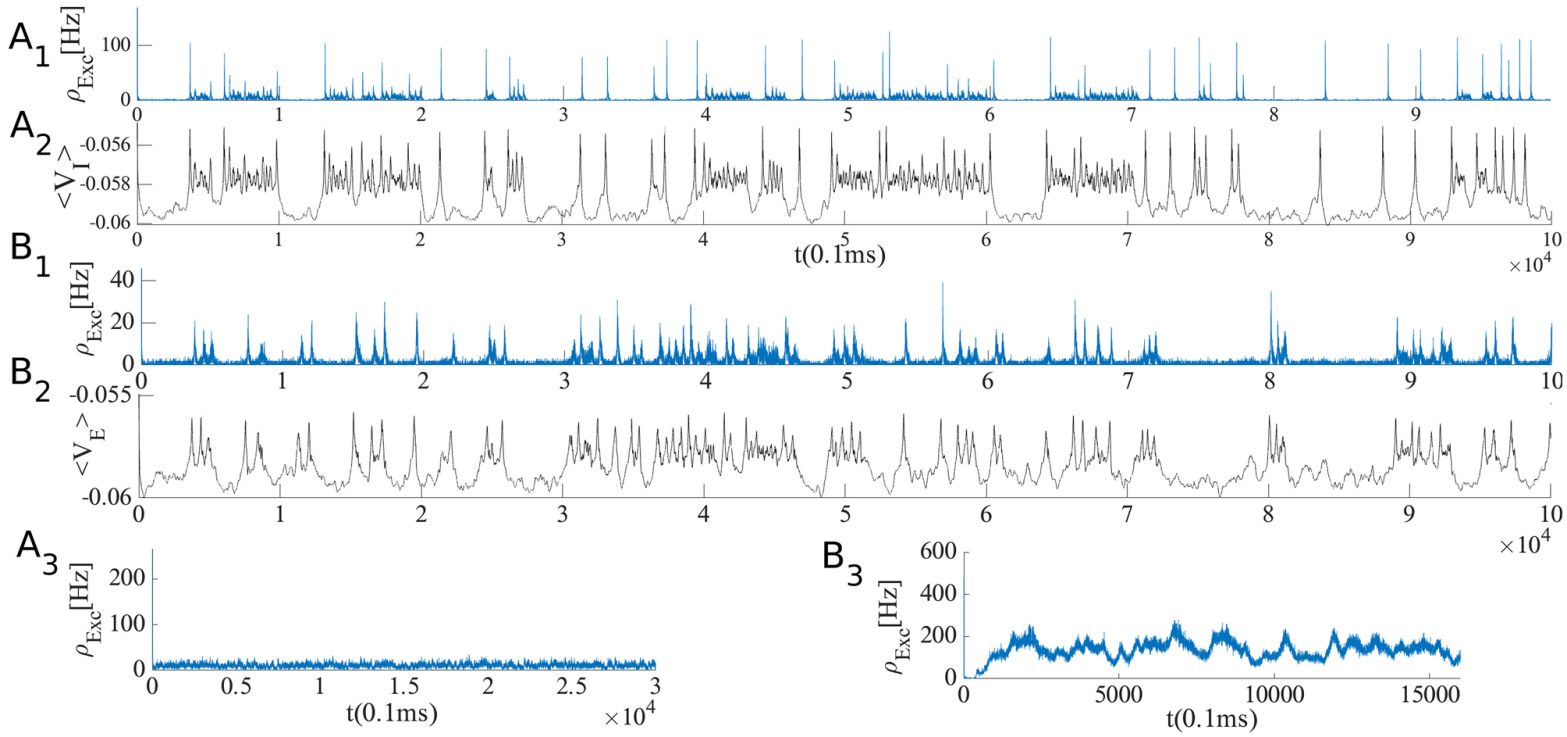
**A1** Avalanches close to the BT point in a system with $W_{II} = W_{EI} = 2 , W_{IE}= 0.75$ , $\rho _{Ext}^{inh} = 150 Hz$ $W_{EE} = 0.615$ and $\rho _{Ext}^{exc}= 218 Hz$. **B1** Avalanches close to the BT point in a system with $W_{EI} = 1.5 ,W_{II}=2 , W_{IE}=0.75$ , $\rho _{Ext}^{inh}$= 150Hz , $\rho _{Ext}^{exc}$ = 230Hz, $W_{EE} = 0.52$ (**A**$_2$) Average membrane potential of inhibitory population shows high fluctuation in the avalanches period and two distinct level of polarization. **B**$_2$ Average membrane potential of excitatory population shows high fluctuation in the avalanches period. In the quiescent state due to excess external current to the excitatory pool the average membrane potential of the excitatory population is slightly higher than the inhibitory one. **A**$_3$ same as **A**$_1$ but with $W_{EE} = 0.61$ and $\rho _{Ext}^{exc} = 223 Hz$. **B**$_3$ same as **B**$_1$ but with $W_{EE} = 0.5$

Figure 13A shows avalanche characteristics of activity in parameter regime on the left of the BT point with the limit cycle solution very close to the origin (region 3 in Fig. 11). Finite size fluctuation leads to switch between these two states. In Fig. 13A$_3$, $W_{EE}$ is higher and $\rho _{Ext}^E$ is slightly lower than the previous case and the system is located in the region with a fixed point in the low firing regime which is stable because of the high value of $W_{II}$ which corresponds to region (5) in Fig. 10. Figure 13B shows avalanche dynamics on the right side of the BT point with an unstable fixed point in the linear section (region (4) in Fig. 11). In both sets of figures increasing $W_{EE}$ moves the system out of the avalanche region with the difference that the fixed point at the linear section is stable in the first case and unstable in the second. Therefore, the nearby regime of activity in the first case (13A$_3$) is a non-oscillatory inhomogeneous Poisson firing state while the corresponding regime near the second case is oscillatory (Fig. 13B$_3$).

#### Stability analysis of fixed points in the linear regime

As we have seen in the last section, close to the BT point there exist regions in which there is a low fixed point at the intersection of the semi-linear sections of nullclines. The stability of the fixed point at the intersection of two nullclines is determined by the Jacobian matrix of the linearized system,

36 $$\begin{aligned} A = \begin{pmatrix} -1 + \dfrac{\partial f}{\partial E} &{} -|\dfrac{\partial f}{\partial I}|\\ \dfrac{\partial g}{\partial E} &{} -1-|\dfrac{\partial g}{\partial I} |\end{pmatrix} \end{aligned}$$

Linear segments intersect if $y_{inh} < y_{exc}$ and $s_{exc} >s_{inh}$ or $y_{inh} > y_{exc}$ and $s_{exc} <s_{inh}$. When the slope and *y*-intercepts are equal, the Jacobian at the point of intersection is

37 $$\begin{aligned} A = \begin{pmatrix} a - \mu &{} - (b -\dfrac{b}{a}\mu ) \\ a &{} -b \end{pmatrix} \end{aligned}$$

with $$\mu = a \dfrac{\rho _{Ext}^E-\rho _{Ext}^I}{d-\rho _{Ext}^I}$$ .

$$a= \dfrac{\partial g}{\partial E} = \alpha ' W_{IE}K_{IE} = \alpha W_{EE}k_{EE}-1+\mu$$

$$d = \dfrac{g_{leak}(V_{rest} - V_{th}) }{\tau * g_{exc}^0*(V_{th}-V_{Rexc})}$$

$$b= 1 + |\dfrac{\partial g}{\partial I} |= 1 + \beta ' W_{II}K_{II} = (1-\dfrac{\mu }{a})^{-1}[\beta W_{EI}k_{EI}]$$

$$\alpha = g_{exc}^0 \tau _{exc} \dfrac{(V_{th} - V_{Rexc})}{\sqrt{2\pi } \sigma _V^{Ex}}$$

$$\alpha ' = g_{exc}^0 \tau _{exc} \dfrac{(V_{th} - V_{Rexc})}{\sqrt{2\pi } \sigma _V^{Inh}}$$

$$\beta = g_{inh}^0 \tau _{inh} \dfrac{(V_{th} - V_{Rinh})}{\sqrt{2\pi } \sigma _V^{Exc}}$$

$$\beta ' = g_{inh}^0 \tau _{inh} \dfrac{(V_{th} - V_{Rinh})}{\sqrt{2\pi } \sigma _V^{Inh}}$$

$$\alpha * \beta ' = \beta * \alpha '$$

Because external excitatory input to the excitatory population is greater than to the inhibitory population and inhibitory connections are assumed to be local, $\mu$ is slightly positive. Define $E= \rho _E- \rho _E^p$ and $I = \rho _I - \rho _I^p$, where $\rho _I^p$ and $\rho _E^p$ is the fixed point location at the linear poisson regime with $\rho _I^p \approx \dfrac{b}{a} \rho _E^p$.

At $\mu = 0$ the eigenvalues of *A* are 0 and $a-b$ with corresponding eigenvectors $u_1 =(\dfrac{b}{a} ,1)$ and $u_2 =(1,1)$. By coordinate transformation to $u_1$ and $u_2$ coordinates, we can write down the dynamics in the decoupled system as

38 $$\begin{aligned} \dot{u} = \begin{pmatrix} 0 &{} 0 \\ 0 &{} a-b \end{pmatrix} u \end{aligned}$$

where,

39 $$\begin{aligned} u = \begin{pmatrix} \dfrac{b}{a} &{} 1 \\ 1 &{} 1 \end{pmatrix}^{-1} \begin{bmatrix} E \\ I \\ \end{bmatrix} = \dfrac{a}{a-b} \begin{bmatrix} I-E \\ E- \dfrac{b}{a}I \\ \end{bmatrix} \end{aligned}$$

with the transformed initial condition

$$\begin{aligned} u_0 = \dfrac{a}{a-b} \begin{bmatrix} I_0 -E_0 \\ E_0 - \dfrac{b}{a}I_0 \\ \end{bmatrix} \end{aligned}$$

which has the following solution in *u* coordinates

40 $$\begin{aligned} u(t) = \dfrac{a}{a-b} \begin{bmatrix} I_0 -E_0 \\ ( E_0 - \dfrac{b}{a}I_0) e^{(a-b)t} \\ \end{bmatrix} \end{aligned}$$

Back into (*E*, *I*) coordinates:

41 $$\begin{aligned} \begin{bmatrix} E(t) \\ I(t) \\ \end{bmatrix} = \dfrac{a}{a-b} ( I_0 -E_0) \begin{bmatrix} \dfrac{b}{a} \\ 1 \\ \end{bmatrix} + \dfrac{a}{a-b}( E_0 - \dfrac{b}{a}I_0) e^{(a-b)t} \begin{bmatrix} 1 \\ 1 \\ \end{bmatrix} \end{aligned}$$

So for this linear system, when $a-b <0$, the initial imbalance of excitatory and inhibitory input leads to a stationary relation of the form $E = \dfrac{b}{a} I$. Now, consider the case in which the linearized nullcline slopes are slightly different with the Jacobian

42 $$\begin{aligned} A = \begin{pmatrix} a - \mu &{} - (b +\epsilon ) \\ a &{} -b \end{pmatrix} \end{aligned}$$

**Fig. 14:**
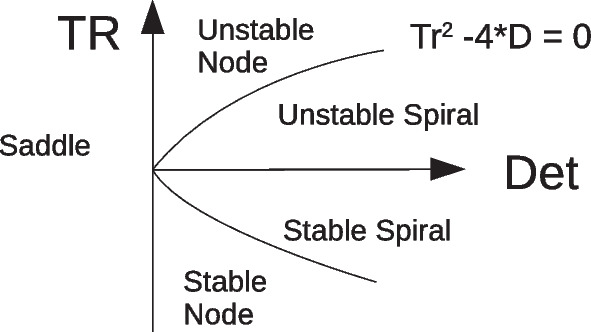
Stability of fixed points in the linear regime based on values of trace and determinant of the Jacobian

Here $TR= \lambda _1 + \lambda _2 = (a-b)-\mu$ and $det= \lambda _1 \lambda _2= a\epsilon + \mu b$. Based on the sign of determinant and trace of the Jacobian at the fixed point, stability is determined (Fig. 14). Under the condition that $b + \mu >a$ and $\epsilon > -\dfrac{b}{a}\mu$, both eigenvalues are negative : $\lambda _1 = \dfrac{b\mu - a\epsilon }{b-a}$ and $\lambda _2 = (a-b) + \dfrac{2a(\epsilon -\mu )}{a-b}$. We also have $|\lambda _1 |<< |\lambda _2 |$ for small differences in the slopes. Eigenvectors corresponding to these eigenvalues are

$$\begin{aligned}&u_1 = ( \dfrac{b}{a} +\lambda _1 , 1) \\&u_2 = (1 + \lambda _2 , 1) \end{aligned}$$

Therefore, the dynamics in the linear regime can be projected to the slow stable manifold $u_1$. One can approximately write down the evolution of the rates as in Eq. 41.

$\epsilon > -\dfrac{b}{a}\mu$ corresponds to the case that the slope of the excitatory nullcline is higher than of the inhibitory nullcline (stronger inhibitory feedback $W_{EI}W_{IE} > W_{II}W_{EE}$ ) and the *y*-intercept of the excitatory nullcline is slightly lower, i.e. stronger external excitatory input to the excitatory population than to the inhibitory one. Moreover, this is the case when $W_{II}$ is high enough to guarantee the $b>a$ condition. When all these requirements are met, the fixed point in the linear segment is stable and we observe an asynchronous low to medium firing state as in Fig. 8 and Fig. ??. Around this regime, an increase in $W_{EE}$ will increase $\mu$ and a change in $\rho _{Ext}^E$ moves the fixed point along the linear section. The intersection in the linear regime transcends to higher rates by increasing $W_{EE}$. This lets the determinant decrease while the trace increases, which eventually destabilizes the fixed point. In the vicinity of the low BT point, based on the value of $W_{II}$, in the linear section either a weakly stable or a weakly unstable fixed point surrounded by a limit cycle appears. In both cases, the eigenvalue close to zero with eigenvector *u*1 governs the slow dynamics around these points.

Consider the case of imaginary eigenvalues of the Jacobian, $\lambda _{\pm } = \sigma \pm i\omega$ with eigenvectors $v_{\pm } = v_r \pm v_i$, which satisfy

$$\begin{aligned}&A [ v_r v_i] = [v_r v_i] \begin{pmatrix} \sigma &{} \omega \\ -\omega &{} \sigma \end{pmatrix} \end{aligned}$$

By defining the tranformation matrix $T = [ v_r v_i]$, the linearized matrix is $$Q = T^{-1}AT = \begin{pmatrix} \sigma &{} \omega \\ -\omega &{} \sigma \end{pmatrix}$$ and the solution of the linear system is of the form

$$\begin{aligned} e^{At} x_0 = T e^{\sigma t}\begin{pmatrix} cos(\omega t) &{} sin(\omega t) \\ - sin(\omega t) &{} cos(\omega t) \end{pmatrix} T^{-1} x_0 \end{aligned}$$

By using the coordinate transformation $u = T^{-1}x$, we can write the evolution $\dot{u} = Qu$ with $u_0 = T^{-1}x_0$. The linearized dynamic predicts damped oscillations of frequency $\omega =\sqrt{det - \dfrac{Tr^2}{4}}$ when $\sigma <0$ and at the Hopf bifurcation point when $\sigma =0$ the frequency of oscillations will be $\omega =\sqrt{det_{H}}$. At the nullcline intersections of linear segments close to the Hopf bifurcation, the oscillation frequency is close to the imaginary part of the eigenvalues: $\sqrt{det - \dfrac{Tr^2}{4}}$.

Along the slow manifold, the inhibitory and excitatory rates vary linearly as $I = \dfrac{a}{b} E \approx \dfrac{k_{ee}W_{ee}}{k_{ei}W_{ei}} E$. This relation balances the average current for each population. Therefore, near the BT bifurcation point, the dynamic of slow field, $E-I$, can be written as

43 $$\begin{aligned} \dfrac{d(E-I)}{dt} = \epsilon (E-I) + c (1-\dfrac{a}{b})^{-1}(E-I)^2 \dfrac{1}{\sqrt{N}}(1-\dfrac{a}{b})^{\dfrac{1}{2}}(E+I)^{\dfrac{1}{2}} \eta (t) \end{aligned}$$

where $\epsilon$ is close to zero, the first nonlinear term of the Taylor expansion has been taken into account and $\eta (t)$ is a white noise added to the microscopic equation based on the Poisson firing assumption.

#### Characteristics of avalanches

**Fig. 15:**
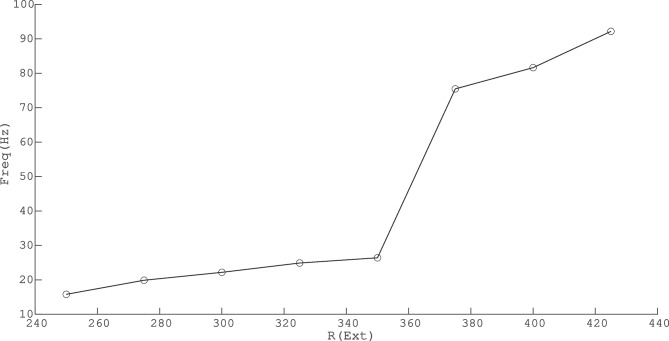
Frequency of avalanches and oscillatory activity increase by input strength. In lower values of excitatory external input, the limit cycle solution is very close to the origin and the system shows avalanches. By increasing the external drive, the limit cycle moves away from the origin (quiescent state) and becomes stable. Oscillations have a higher frequency at higher external input rates with semi-linear relations in both regions

For the values of $W_{EE}$ near the BT point at the low firing rate, there exists a range of external input strength for which the firing pattern is quasi-periodic with excitatory avalanches followed by inhibitory ones. The mean escape time from the basin of attraction of the quiescent fixed point reduces when the external input increases, and thus, the frequency of avalanches increases. Further increase of external input leads to stability loss of the quiescent state and appearance of higher frequency oscillations in the medium range of rates (see Fig. 15).

**Fig. 16:**
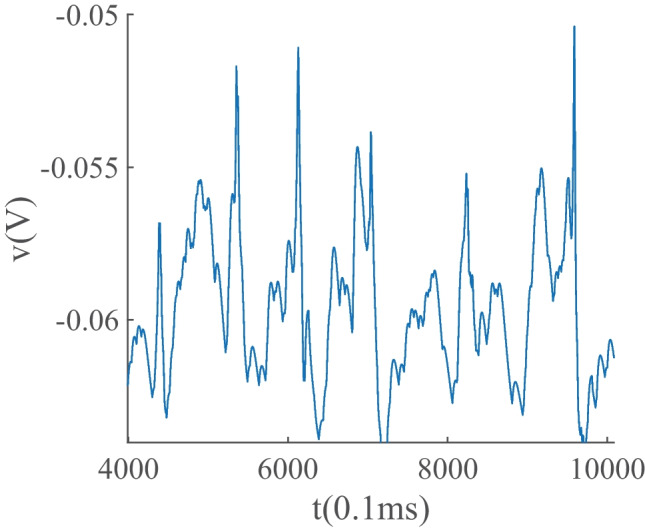
Membrane potential track of a single neuron during avalanche dynamic of Fig. 13. Avalanches at population level can be seen as periods of rising potential in the individual neurons which are sustained longer than avalanche range due to slow synaptic decay. Individual neurons do not fire in every single avalanche

In the avalanche regime, the membrane potential shows sub-threshold oscillations as can be seen in Fig. 13. In the down phase of the cycle, neurons stay near the resting potential while at the up-state they reside closer to the threshold, but at a distance that permits high variability of firing. The membrane potential of a single neuron is depicted in Fig. 16, which shows aperiodic firing and up-down states of membrane potential.

**Fig. 17:**
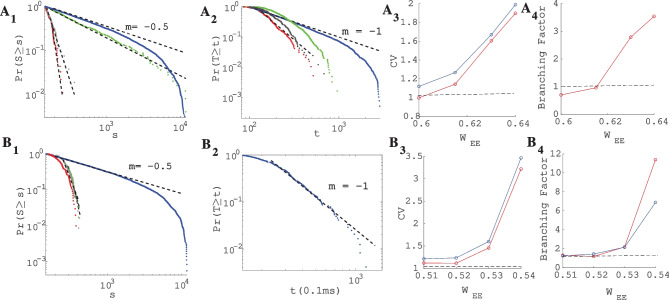
**A** Same as Fig. 13A with $W_{EI} = 2 ,W_{II}=2 , W_{IE}=0.75$, $\rho _{Ext}^{inh}$ = 150Hz, $\rho _{Ext}^{exc}$ = 230Hz with different values of $W_{EE} \in (0.6,0.65)$. Cumulative distribution function of avalanche sizes (**A**$_1$) and duration (**A**$_2$) in log-log plot with linear fit. Red curve ($W_{EE}$=0.64), black ($W_{EE}$ = 0.63), blue ($W_{EE}$= 0.615) and green ($W_{EE}$ = 0.6). (**A**$_3$) Branching ratio and (**A**$_4$) CV of firing time intervals of individual neurons (red for the excitatory neurons and blue for the inhibitory ones). **B** Characteristics of avalanches for the model with $W_{EI} = 1.5 ,W_{II}=2 , W_{IE}=0.75$ , $\rho _{Ext}^{inh}$= 150Hz , $\rho _{Ext}^{exc}$ = 230Hz and $W_{EE} \in (0.516,0.54)$ as in Fig. 13B . Cumulative distribution function of avalanche sizes (**B**$_1$) and duration (**B**$_2$) in log-log plot with linear fit. Green curve ($W_{EE}$ = 0.54), black ($W_{EE}$ = 0.53), blue ($W_{EE}$ = 0.52) and red ($W_{EE}$ = 0.51). (**B**$_3$) Branching ratio and (**B**$_4$) CV of interspike time intervals of individual neurons (red for the excitatory neurons and blue for the inhibitory ones)

While avalanches occur quasiperiodically, in most of them only a fraction of neurons fire. As shown in (Fig. 17A$_3$ and B$_3$) neurons fire with CV close to one in the lower $W_{EE}$ regime, close to the BT point. Variability in the size of avalanches is another interesting item to investigate.

We define avalanches as bursts of activity higher than a threshold which is around 0.2*Hz* corresponding to random background activity driven by external noise at the quiescent state. Choosing a small threshold value in a way to exclude single neurons firing while observing scaling in large avalanche sizes does not affect asymptotic exponents as it has no effect on identifying the avalanches of large sizes and their relative frequencies. Using the algorithm introduced in Clauset et al. (2009), we fit the power-law distribution to avalanches size and duration with the maximum likelihood estimator(MLE) algorithm adjusting for $s_{min}$ which leads to the best power-law fit with the lowest Kolmogorov-Smirnov (KS) measure. We check the goodness of the fit and also compute directly the MLE from the following formula:

44 $$\begin{aligned} \alpha ^ * = 1 + N (\sum _i log( \dfrac{s_i}{s_{min}}))^{-1} \end{aligned}$$

**Fig. 18:**
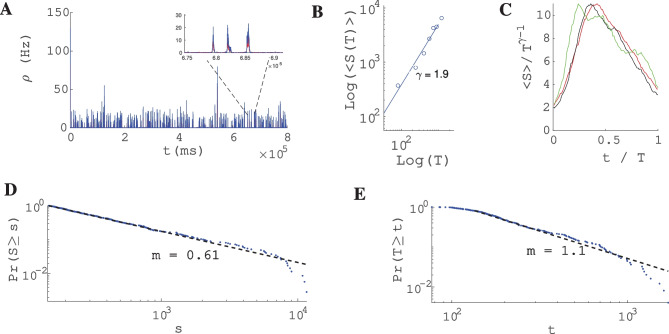
**A** Excitatory (red) and Inhibitory (blue) population rates in the avalanches regime. Inhibitory instantaneous rate is proportional to the excitatory rate which leads to tight temporal balance. **B** Average size vs. average duration of avalanches. **C** Temporal profile of three sets of avalanches with different duration. **D** The cumulative distribution function of inhibitory avalanches size. (KS = 0.056) **E** Cumulative distribution function of inhibitory avalanche duration. (KS = 0.068)

The size distribution of avalanches has a longer tail approaching the BT point. It follows a power-law probability density function (PDF) for avalanche size $P(S) \propto S^{-\tau }$ with slope $\tau = 1.5$ close to the BT point, see Fig. 17A$_1$ and B$_1$. Further away from the critical point, avalanches have characteristic average size and their size probability density moves away from the power-law distribution. Furthermore, the probability distribution for the duration of avalanches follows a power law with an exponent close to $-\alpha = -2$ near the BT point (see Fig. 17$A_2$ and B$_2$). To further analyze the criticality hypothesis, we have computed the avalanche shape collapse and scaling relation (see Fig. 18). Average size versus average duration of avalanches obeys a power-law with $\langle s\rangle = \langle T\rangle ^{\dfrac{1}{\sigma \nu z}}$ linked by a scaling relation $\dfrac{\alpha -1}{\tau -1} = \dfrac{1}{\sigma \nu z} \approx$2 between exponents. In addition, the mean temporal profile of avalanches follows a scaling form ,

45 $$\begin{aligned} S(t, T) \sim T^{1/\sigma \nu z -1} F(t/T) \end{aligned}$$

Furthermore, size and duration of avalanches in the inhibitory population obey power law scaling as well (see Fig. 18D-E). The critical exponents and the scaling relations among them suggest that the critical phase transition in our system belongs to the directed percolation universality class.

The branching ratio can be defined as the average number of postsynaptic neurons of a specific neuron that fire by receiving the synaptic current from that neuron. The branching ratio can be an indicator of scale-free avalanche dynamics. When inhibition and excitation are balanced and the system resides near a quiescent state, the branching parameter stays close to, but below one which is an indicator of stronger inhibitory feedback. As can be seen in Fig. 17, this value is lower in the paramter regime close to the BT point and becomes $>1$ further away from it.

Here, we assume that by synchronous activation of $n_E$ neurons the postsynaptic neurons which are connected to these neurons will receive both excitatory and inhibitory currents caused by the synchronous input. Each neuron receives a fraction $k_{EE}$ of excitatory and $k_{EI}$ of inhibitory currents produced by active neurons. The average potential change among neurons will be

46 $$\begin{aligned} \langle \Delta V \rangle =&\langle k_{EE} n_E\rangle \dfrac{1}{C} g_0 w_{EE} \tau (V_{Rexc}- V_{E} ) + \langle k_{EI} n_I \rangle \dfrac{1}{C} g_0 w_E \tau (V_{Rexc}- V_{E} ) \end{aligned}$$

Close to the bifurcation point, there exists a tight dynamic balance between excitatory and inhibitory rates, following Eq. 41, which sets $\langle \Delta V \rangle =0$. Based on the assumption that neurons fire with Poisson statistics, we can write the variance of the potential change in the postsynaptic neuron pool as

47 $$\begin{aligned} \langle \Delta V ^2\rangle = \tau ^2 g_0^2 ( \langle k_{EE}n_E\rangle w_{EE}^2 (v_{Rexc} - V_{th})^2 + \langle k_{EI}n_I\rangle (w_{EI})^2( v_{Rinh} - V_{th})^2 ) \end{aligned}$$

On the other hand, the number of postsynaptic neurons that fire by receiving an increase in voltage of value $\Delta V$ is

48 $$\begin{aligned} \sigma = N_{Exc} \int _{V_{th} - \Delta V}^{V_{th}} P(V,t=\infty )\approx&- \dfrac{N_{exc}\Delta V^2}{2}\dfrac{\partial p(v^E ,t=\infty )}{\partial v} \mid _{v^E = V_{th}} \end{aligned}$$

From Eq. S.10 in SM , for the stationary probability density we have

49 $$\begin{aligned}&\dfrac{\partial p(v^E ,t=\infty )}{\partial v}\mid _{v^E = V_{th}} = - \dfrac{2 C^2\rho _{exc}}{ D_e( v_{Rexc} - V_{th})^2 + D_i( v_{Rinh} - V_{th})^2} \end{aligned}$$

Inserting Eq. 49 in Eq. 48 and averaging $\sigma$ over different realizations of the synchronous firing using Eq. 47 and dividing by $\langle n_E \rangle$ leads to

50 $$\begin{aligned} \sigma ^E \approx \dfrac{ \tau ^2 g_0^2 [w_{EE}^2 \rho _{exc}^{st} (v_{Rexc} - V_{th})^2 + \rho _{exc}^{st} \dfrac{\langle n_I\rangle }{ \langle n_E \rangle } w_{EI}^2( v_{Rinh} - V_{th})^2 ]}{ D_e( v_{Rexc} - V_{th})^2 + D_i( v_{Rinh} - V_{th})^2 } \end{aligned}$$

The average number of active inhibitory and excitatory neurons $\langle n_I\rangle$ and $\langle n_E \rangle$, relates to stationary rates as $\dfrac{\langle n_I \rangle }{\langle n_E \rangle } = \dfrac{ \rho _I}{\rho _E}$. Inserting this relation into Eq. 50, we find out that the branching ratio is close to one near the BT point. Because of slightly stronger inhibitory feedback, it is slightly below one.

Excitatory neurons stay in a low firing regime with average membrane potential close to the middle point between the firing threshold and the resting-state potential, i.e., at $V \sim -57mv$. At this point, a sufficient fraction of neurons is close to the threshold, whose activation can cause a series of firing. On the other hand, inhibitory neurons, which have a lower stationary membrane potential because of lower external input, provide negative feedback with a delay that depends on the resting initial state and the strength of the connection between inhibitory and excitatory sub-networks. The dynamic balance of excitation and inhibition in the linear UP state leads to critical behavior. As average currents to the cells are balanced far from the firing threshold, fluctuations in these currents have a larger effect and therefore, the size of events and their durations are more variable.

**Fig. 19:**
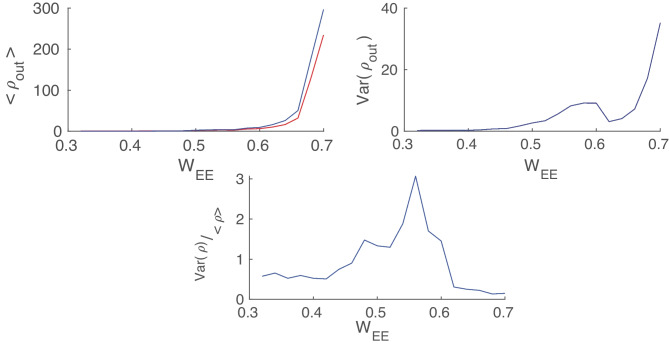
Stationary population rates (**A**) (Blue for Inh. and Red for Exc.), variance (**B**) and normalized variance (**C**) for EI population vs. $W_{EE}$ at the fixed value of $\rho _{Ext} =250Hz$. Other parameters were set to $W_{IE}= 0.75$, $\rho _{Inh}=150Hz$, $W_{II}=W_{EI}=2$

**Fig. 20:**
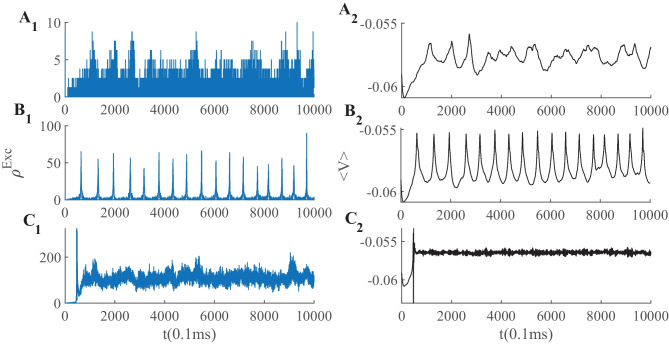
Excitatory population rate (Left plots) and average membrane potential (Right plots) when the system is slightly below the Hopf bifurcation point (**A**), slightly above Hopf bifurcation point (**B**), or after the saddle-node bifurcation point (**C**) corresponding to the states in Fig. 19 with the same parameters

Moreover, let us consider the onset of avalanche dynamics in the EI population receiving external input with fixed rates by selecting $W_{EE}$ as the only dynamic parameter (see Fig. 19). By increasing $W_{EE}$, a second-order phase transition happens at the Hopf bifurcation. Around this value, the normalized variance of the population rate is maximized and oscillations appear in the system. In Fig. 20, this happens at the value $W_{EE} \approx 0.57$. Further increase of $W_{EE}$ results in the saddle-node bifurcation which produces a stable high firing rate state at values around $W_{EE} \approx 0.67$.

Although the activity is noise-driven, the state of the system depends on synaptic weights, which determine the response to the external input. There must be a self-organizing mechanism, which in a wider range of input strengths and initial configurations of synaptic weights tunes the system close to the BT point.

## Discussion

We have seen that in a large sparse network of spiking neurons the input to the cells in the state of asynchronous firing is Poisson and investigated conditions on Poisson firing at the single neuron level. We chose the conductance-based leaky integrate and fire model to take the strong dependence of the inhibitory postsynaptic current on the voltage level into consideration. Next, we introduced linearization of the neuron gain function in the Poisson firing regime and presented a linear Poisson neuron model which we used to analyze interconnected networks of excitatory and the inhibitory neurons.

The network of spiking neurons with the assumptions of homogeneity, large size, and sparse connectivity can be modeled by the dynamics of the mean field. The excitatory and inhibitory mean-field equations are a set of nonlinear equations with free parameters including the average synaptic strength between different types of neurons. Taking a set of these free parameters as control parameters of the model one can analyze the bifurcation patterns in the system. Here, we chose the excitatory external drive and the synaptic weight from excitatory to excitatory neurons as control parameters. The latter regulates the strength of the inhibitory feedback in the local population and the former controls the level of forced activity from other populations. The qualitative picture of the bifurcation patterns does not change by the choice of different synaptic weights as control parameter. In analyzing the bifurcation diagram, we are mainly interested in the loss of stability of the quiescent state. This can happen through a saddle-node or a Hopf bifurcation by either increasing the external drive or $W_{EE}$. At a certain point called the Bogdanov-Takens point, the saddle-node and Hopf bifurcation lines meet. Near this point there is a tight balance of the inhibitory and the excitatory average currents to the cells. This balance cancels out much of the high amplitude excitatory and inhibitory currents to each cell and causes the average membrane potential of the neurons in the population to stay away from the threshold. In this regime, the activity of the spiking neurons is fluctuation driven which makes the firing time intervals highly variable. In this case, the statistics of the firing is close to a Poisson point process matching the experimental findings. On the other hand, the balance of excitation and inhibition leads to avalanche style dynamics near the BT point. Slow oscillations emerge at the Hopf bifurcation line and through a saddle-node bifurcation, a pair of low firing stable and unstable fixed points comes into existence.

The next step after identifying the operating dynamical regime that produces the desired output is to investigate mechanisms that can tune the parameters of the system at the desired region of the phase space. We shall investigate the self-organization by spike-timing dependent plasticity and short term synaptic depression in another article (Ehsani and Jost (2022)). The former tunes the overall strength of excitatory and inhibitory pathways to be close to a balanced regime of these currents and the latter, which is based on the finite amount of resources in brain areas, acts as an adaptive mechanism that tunes micro populations of neurons subjected to fluctuating external inputs to attain the balance in a wider range of external input strengths.

Our analysis in this article is restricted to the case of homogeneous neuronal network. In biological neuronal network there is high level of modularity in large scale connectivity maps and inhomogeneity in number and strength of connections among different neurons. Therefore, effect of modular hierarchical inhomogeneous structure on the critical behaviour of the system requires further investigation. Wardak and Gong (2022) studied criticality in excitatory networks with heavy-tailed probabilty distribution for synaptic connections and reported extended critcal regimes between quiescent and active states. Kuśmierz et al. (2020) showed a continuous transition to chaos in an excitatory network with power-law distributed synaptic weights. It is of interest to analytically investigate how heavy-tailed synaptic weights affect the critical region surrounding the BT point in EI networks. In addition, we have only studied the single EI population dynamic in this work. Analyzing weakly interconnected neuronal population and coarse grained field equations near BT point and investigating the types of solutions that emerge in the continuum limit is another extension of the current work. In Ehsani and Jost (2022), we introduced a phonological stochastic field equation model for EI population near the BT point. However, extensive analysis of types of solutions of these sets of equations is required to shed light on large scale behaviour of a system which is locally tuned at the BT point

## Supplementary Information

Below is the link to the electronic supplementary material.

Supplementary file1 (PDF 442 KB)

## Data Availability

The original contributions presented in the study are included in the article/supplementary material, further inquiries can be directed to the corresponding author.

## Code availability

Code availability :The code used in this work is available for download through github under the following link:
