# Supplementary material for: Scale free avalanches in excitatory-inhibitory populations of spiking neurons with conductance based synaptic currents
Source: J Comput Neurosci. 2022 Oct 25;51(1):149–72. doi: 10.1007/s10827-022-00838-4 (PMC9840601; doi:10.1007/s10827-022-00838-4)
Supplement: Supplementary file 1 — Supplementary file1 (PDF 442 KB) [file 10827_2022_838_MOESM1_ESM.pdf]

Supplementary Material for : Scale Free Avalanches in Excitatory-Inhibitory Populations of Spiking Neurons with Conductance Based Synaptic Currents

Masud Ehsani[1*] and Jürgen Jost[1,2]

[1*] Max Planck Institute for Mathematics in Sciences, Inselstr.22, Leipzig, 04103, Saxony, Germany .
[2]Santa Fe Institute, 1399 Hyde Park Rd, Santa Fe, NM 87501, United States .

*Corresponding author(s). E-mail(s): masud.ehsani@mis.mpg.de;
Contributing authors: jjost@mis.mpg.de;

1 Linear Poisson Neuron

1.1 Diffusion Approximation

The Poisson input in the limit of a high firing rate and small synaptic weights can be approximated by a diffusion process. Suppose, in the time interval $[t, t+dt]$, $N(t, t+dt)$ excitatory spikes arrive at the cell each with synaptic strength w_e. As the spike arrival is a Poisson process with the rate λ the distribution of $N(t, t+dt)$ is Poisson and all the cumulants of the random variable N are equal to λdt. This leads to the following cumulant for $I(t, t+dt) = w_e N(t, t+dt)$:

$$\begin{aligned}
\kappa_1 &= \langle I_{t,t+dt} \rangle dt = w_e \lambda dt \\
\kappa_2 &= Var(I_{t,t+dt}) = w_e^2 \lambda dt \\
\kappa_3 &= w_e^3 \lambda dt
\end{aligned} \tag{1}$$

Higher cumulants can be ignored if we assume that $w_e^3 \lambda$ goes to zero in the limit of a high number of afferent inputs. In theory, this can be achieved by

assuming weights to scale as $w_e = \frac{W}{\sqrt{k}}$ where k is the number of presynaptic neurons. In this case, $\lambda \sim O(k)$ and the average excitatory and inhibitory currents are each of order $O(\sqrt{k})$, the variance of the current is of $O(1)$ and higher cumulants vanish in the limit of large k. In this case, one can take $I(t, t+dt)$ as a Gaussian random variable with mean and variance given by the above equation. It can also be written as

$$I(t)dt = w_e \lambda dt + w_e \sqrt{\lambda} dW_t \tag{2}$$

where W_t is a Wiener process.

In the conductance based model the input to the cell causes a change in the conductance. As the input is stochastic the conductance is also a stochastic variable which can be written as

$$g(t) = \int_{-\infty}^{t} g_0 e^{-\frac{t-s}{\tau}} I(s)ds = \int_{-\infty}^{t} e^{-\frac{t-s}{\tau}} g_0 w_e \lambda ds + \int_{-\infty}^{t} e^{-\frac{t-s}{\tau}} g_0 w_e \sqrt{\lambda} dW_s \tag{3}$$

The second term is the integral of a Wiener process with exponential kernel. For a stochastic process $Y_t = \int_{-\inf}^{t} f(s)dW_s$, we can easily verify that:

$$\begin{aligned} \langle Y_t \rangle =& 0 \\ \langle Y_t^2 \rangle =& \int_{-\inf}^{t} f(s)^2 ds \end{aligned} \tag{4}$$

If we consider a stationary and homogeneous Poisson process as the input then $g(t)$ will also attain a stationary probability distribution. Using the above equation, mean and variance of $g(t)$ reach the limits

$$\begin{aligned} \langle g \rangle =& \tau g_0 w_e \lambda \\ Var(g) =& \frac{\tau g_0^2 w_e^2 \lambda}{2} \end{aligned} \tag{5}$$

With the above scaling of the weights, higher cumulants vanish and one can assume that $g(t)$ reaches a stationary Gaussian probability distribution with mean and variance as above. The covariance of this process can be derived by direct multiplication and averaging over the noise terms of $g(t)$ and $g(t')$ from Equation 5 to reach the following equation when $t' > t$:

$$\langle g(t)g(t') \rangle - \langle g(t) \rangle \langle g(t') \rangle = Var(g) e^{-\frac{(t'-t)}{\tau}} \tag{6}$$

The same procedure applies to inhibitory currents. Figure 1 shows statistics of conductance $g(t)$ and the validity of diffusion approximation. If we assume

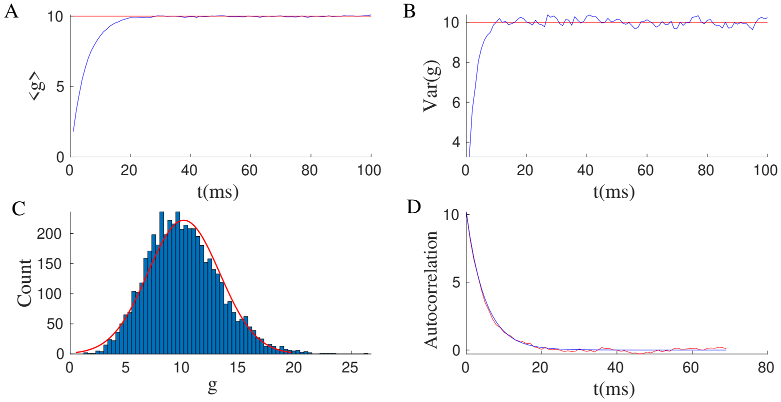

Fig. 1: Mean (A), Variance (B), stationary probability distribution (C), and auto-correlation (D) of the conductance $g(t)$ of a neuron receiving Poisson input. The red lines are the values calculated by the diffusion approximation and the red curve in the bottom left plot is the Gaussian distribution with the mean and variance as derived in the text. The auto-correlation matches Equation 6. This figure shows that the diffusion approximation is valid.

that the synaptic time scale τ is very small in comparison to the membrane potential time scale, then one can ignore the cross correlation and consider $Y_t = g(t) - \langle g \rangle$ as Gaussian white noise. In this limit,

$$\lim_{\tau \to 0} \langle Y_t Y_s \rangle = 2\tau var(g) \lim_{\tau \to 0} \frac{e^{-\dfrac{(s-t)}{\tau}}}{2\tau} = 2\tau var(g)\delta(t-s) \tag{7}$$

This leads to a stochastic differential equation for the membrane potential evolution in the conductance-based model, when $v(t) < V_{th}$:

$$\begin{aligned} C\frac{dv(t)}{dt} =& [g_{Leak}(v_{Leak} - v(t))g_0 w_e \lambda_e \tau (v_{Rexc} - v(t))g_0 w_i \lambda_i \tau (v_{Rinh} - v(t))] \\ &+ \xi_{exc}(t)(v_{Rexc} - v(t)) + \xi_{inh}(t)(v(t) - v_{Rinh}) \\ &\equiv [a - bv] + \xi_{exc}(t)(v_{Rexc} - v(t)) + \xi_{inh}(t)(v(t) - v_{Rinh}) \end{aligned} \tag{8}$$

Here $\xi_{exc}(t)$ and $\xi_{inh}(t)$ are purely random Gaussian processes:

$$\begin{aligned} &\langle \xi_{exc}(t) \rangle = \langle \xi_{inh}(t) \rangle = 0 \\ &\langle \xi_{exc}(t)\xi_{exc}(t') \rangle = \tau^2 g_0^2 w_e^2 \lambda_e \delta(t-t') \equiv D_e \delta(t-t') \\ &\langle \xi_{inh}(t)\xi_{inh}(t') \rangle = \tau^2 g_0^2 w_i^2 \lambda_i \delta(t-t') \equiv D_i \delta(t-t'). \end{aligned}$$

The first line of Equation 8 is the deterministic evolution of the potential. When the fixed point of the deterministic term, $v_{det}^{\inf} = V_{st} = \frac{a}{b}$ as defined by Equation 8 is greater than V_{th}, the effect of fluctuations is marginal and the firing of the neuron is governed by the drift term. However, when V_{st} is below the threshold, fluctuations in the input can result in firing of the neuron.

In the following, we calculate the variance and the mean of the membrane potential and the output firing rate of the neuron in the Gaussian approximation. The Fokker-Planck equation corresponding to Eq.8 in the Itô interpretation is

$$\begin{aligned}\frac{\partial p(v,t)}{\partial t} =& -\frac{1}{C}\frac{\partial}{\partial v}[(a-bv)p(v,t)] + \frac{1}{C^2}\frac{D_e}{2}\frac{\partial^2}{\partial v^2}(v_{Rexc}-v(t))^2 p(v,t)\\ &+ \frac{1}{C^2}\frac{D_i}{2}\frac{\partial^2}{\partial v^2}(v_{Rinh}-v(t))^2 p(v,t) \equiv -\frac{\partial}{\partial v}J(v,t) \qquad (9)\end{aligned}$$

with the boundary condition $p(V_{th}, t) = 0$. The density current at $v = V_{th}$ is equivalent to the firing rate, and this current is fed back to the equation at $v = V_r$ resulting in a discontinuity of the membrane potential derivative.

$$J(v_r^+, t) - J(v_r^-, t) = J(v_{th}, t - t_{ref}) \equiv r(t - t_{ref})$$

The stationary probability distribution and firing rate are obtained by solving the equation

$$J_{st}(v) = r_0 \Theta(v - V_r)$$

which results in

$$\begin{aligned}&\frac{1}{C}\{a - bv + \frac{1}{C}D_e(v_{Rexc} - v) + \frac{1}{C}D_i(v_{Rinh} - v)\}p(v)\\ &-\frac{1}{C^2}\{\frac{D_e}{2}(v_{Rexc} - v)^2 + \frac{D_i}{2}(v_{Rinh} - v)^2)\}\frac{dp(v)}{dv} = r \qquad (10)\end{aligned}$$

for $V_r < v < V_{th}$. Together with the normalization requirement $\int_{-\infty}^{V_{th}} p(v)dv + r_0 * t_{ref} = 1$ one can solve equation 10. numerically for both the stationary probability distribution and the stationary firing rate.

When V_{st} is sufficiently smaller than V_{th} , i.e., in the low firing rate regime, we can ignore the non-linearity caused by the threshold and write down the evolution of the mean and variance of the membrane potential as follows (See Fig.2)

$$\frac{d\langle v(t)\rangle}{dt} = \frac{1}{C}(a - b\langle v(t)\rangle)$$

$$\frac{dVar(v,t)}{dt} = -\frac{2b}{C}Var(v,t) + \frac{1}{C^2}[D_e\langle(V_{Rexc} - v(t))^2\rangle + D_i\langle(v(t) - V_{Rinh})^2\rangle]$$
$$= (-\frac{2b}{C} + \frac{D_e + D_i}{C^2})Var(v,t) + \frac{1}{C^2}[D_e(V_{Rexc} - \langle v\rangle)^2 + D_i(V_{Rinh} - \langle v\rangle)^2]$$

This leads to the stationary value for average and variance of the membrane voltage

$$\langle v\rangle_{st} = \frac{a}{b}$$
$$Var(v)_{st} = \frac{1}{2bC - (D_e + D_i)}[D_e(V_{Rexc} - \langle v\rangle_{st})^2 + D_i(\langle v\rangle_{st} - V_{Rinh})^2] \quad (11)$$

In the low firing regime, the stationary probability distribution can be approximated as (see Fig.3):

$$P_{st}(V) = \frac{1}{\sqrt{2\pi}\sigma_{V(st)}}exp(-\frac{(V - \langle V\rangle)^2}{2\sigma^2})$$
$$+ c\delta(V - V_{Rest}) \qquad V < V_{th}$$
$$P_{st}(V) = 0 \qquad V \geq V_{th}$$

The stationary firing rate is derived from equation 10 by plugging in the Gaussian approximation for the stationary potential probability density $P(V, t \to \infty) = N(\langle V\rangle, \sigma_{V(st)})$:

$$r = \frac{1}{C^2}\frac{1}{2}D(V_{th})^2\frac{dp(V)}{dV}_{|V=V_{th}}$$
$$= -\frac{b}{C}\frac{D(V_{th})^2}{D(\langle V\rangle)^2} * \frac{(V_{th} - \langle V\rangle)}{\sigma_{V(st)}\sqrt{2\pi}}exp(-\frac{(V_{th} - \langle V\rangle)^2}{2\sigma^2_{V(st)})})$$
$$\approx \frac{b}{\sqrt{\pi}C}\frac{(V_{th} - \langle V\rangle)}{\sqrt{2}\sigma_{V(st)}}(1 - \frac{(V_{th} - \langle V\rangle)^2}{2\sigma_{V(st)^2}}) \quad (12)$$

We can improve the approximation for the potential distribution and the firing rate by considering the autocorrelation in the conductance. When τ is not negligible but small, we can use the τ expansion method to account for first-order corrections to the Fokker-Planck equation (section 1.1.1).

1.1.1 Tau expansion

The equation $\frac{dv(t)}{dt} = f(v) + \eta(t)g(v)$ where $\eta(t)$ is colored Gaussian noise with correlation

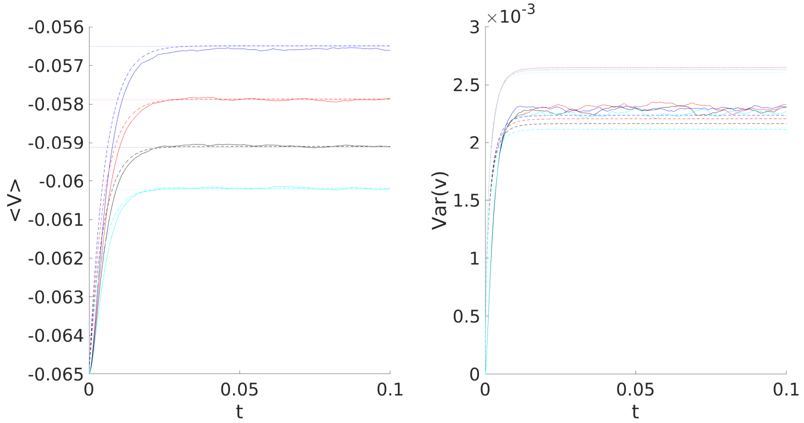

Fig. 2: Evolution of the mean and the variance of the potential distribution of a population of neurons each receiving a fixed equal inhibitory input rate but different excitatory rates. Dashed lines are the trajectories determined from Gaussian noise approximation and the dotted lines are derived from the tau expansion method. The approximation by tau expansion improves the estimation of variance.($\tau_{syn} = 2.5ms$)

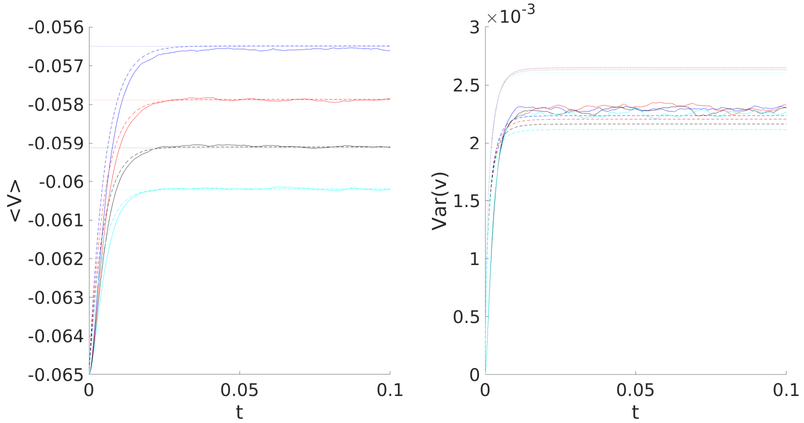

Fig. 3: Membrane potential distribution and Gaussian approximation (Blue lines) for two different sets of inhibitory and excitatory Poisson inputs. The average membrane potential values are $-0.59mv$(Left) and $-0.56mv$(Right)

$$\langle \eta(t)\eta(t') \rangle = \frac{H}{\tau} e^{\frac{-(t-t')}{\tau}}$$

corresponds to the following Fokker-Planck equation derived by expansion with respect to τ :

$$\begin{aligned}\frac{\partial p(v,t)}{\partial t} =& -\frac{\partial}{\partial v}[f(v)p(v,t)] + H\frac{\partial}{\partial v}g(v)\frac{\partial}{\partial v}\{g(v)[1+\tau g(v)(\frac{f(v)}{g(v)})']p(v,t)\}\\ =& -\frac{\partial}{\partial v}[(f(v)+Hg(v)g'(v))p(v,t)] + H\frac{\partial^2}{\partial v^2}\{g(v)^2 p(v,t)\}\\ &-\tau H\frac{\partial}{\partial v}\{g'(v)(g(v)f'(v)-g'(v)f(v))P(v,t)\}\\ &+\tau H\frac{\partial^2}{\partial v^2}\{g(v)(g(v)f'(v)-g'(v)f(v))p(v,t)\}\end{aligned}$$

In our case $H_e = \frac{\tau^2 g_0^2 w_e^2 \lambda_e}{2} = \frac{D_e}{2}$, resulting in:

$$\begin{aligned}\frac{\partial p(v,t)}{\partial t} =& -\frac{1}{C}\frac{\partial}{\partial v}\{(a-bv+F(v))p(v,t)\}\\ &+\frac{D_e}{2C^2}\frac{\partial^2}{\partial v^2}\{G_e(v)p(v,t)\} + \frac{D_i}{2C^2}\frac{\partial^2}{\partial v^2}\{G_i(v)p(v,t)\}\end{aligned} \tag{13}$$

where

$$\begin{aligned}F(v) =& \frac{D_e}{2C}[-2(v_{Rexc}-v(t)) - \frac{\tau}{C}(a-bV_{Rexc})]\\ &+\frac{D_i}{2C}[-2(v_{Rinh}-v(t)) - \frac{\tau}{C}(a-bV_{Rinh})]\\ G_e(v) =& (v_{Rexc}-v(t))^2 + \frac{\tau}{C}(v_{Rexc}-v(t))(a-bV_{Rexc})\\ G_i(v) =& (v_{Rinh}-v(t))^2 + \frac{\tau}{C}(v_{Rinh}-v(t))(a-bV_{Rinh})\end{aligned}$$

Figure 4C shows that these corrections lead to a better approximation of the stationary membrane potential variance in a low firing rate regime. The stationary variance of the membrane potential decreases at higher values of τ_{syn} (Figures 4A and 4B) . Higher rates in excitatory and inhibitory input would lead to lower stationary variance and lower rates(section 1.1.2).

1.1.2 Neuron response in higher values of input rates and synaptic time constant

As it can be seen from Fig.2 , the stationary standard deviation of the membrane potential in the case of Poisson input does not show high sensitivity to input rates when the stationary mean potential is fairly away from the threshold. In Fig.4, increasing the excitatory input rate by 60% causes a 2% increase of $\sigma(V)_{st}$ on average for different values of $\langle v\rangle_{st}$. This can be predicted from

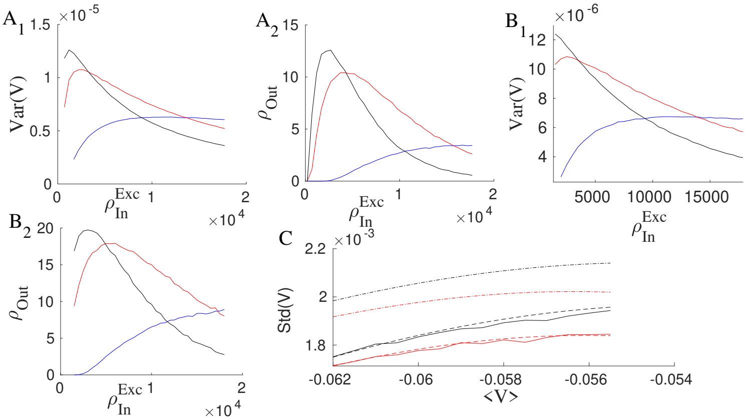

Fig. 4: (A1-A2) Stationary variance of the membrane potential and the output firing rates, when the excitatory rate (x-axis) and inhibitory rates are balanced so that the average membrane potential is at $-57mv$ for three different values of τ_{syn} = [1 (Blue), 3 (Red), 5 (Black)] ms. (B1-B2) Same with average membrane potential tuned at the threshold value $-50mv$. (C) Stationary potential variance for a neuron receiving two different excitatory input rates, 1000Hz(red) and 1600Hz (black), and corresponding inhibitory input, which places the average potential at a specific value shown in the x-axis. Solid lines are simulation results, dashed lines correspond to low firing regimes with tau approximation (Equation 13), and fine dashed lines show Gaussian approximation. ($\tau_{syn} = 2.5ms$)

equation 11 as both the input noise and drift term in the numerator and the denominator depend linearly on input rates. Suppose $\langle v \rangle_{st}$ is fixed for a set of inhibitory and excitatory rates, which means there is a linear relation of the form $\rho_E = \kappa\rho_I + c$ originating from the condition on the fixed stationary average membrane potential. In the limit of high rates, the stationary variance approaches a constant value:

$$Var(v)_{st} = \frac{\sigma^2}{2bC} = \frac{\alpha - \frac{\beta}{\lambda_E}}{\gamma - \frac{\eta}{\lambda_E}}$$

$$\frac{\alpha}{\gamma} = \frac{\tau g_0 (V_{Rexc} - \langle V_{st} \rangle)(\langle V_{st} \rangle - V_{Rinh})}{V_{Rexc} - V_{Rinh}} * [w_E(V_{Rexc} - \langle V_{st} \rangle) + w_I(\langle V_{st} \rangle - V_{Rinh})]$$

$$\frac{\beta}{\eta} = \frac{w_i \tau g_0 (\langle V_{st} \rangle - V_{Leak})(\langle V_{st} \rangle - V_{Rinh})^2}{\langle V_{st} \rangle - V_{leak}} = w_i \tau g_0 (\langle V_{st} \rangle - V_{Rinh})^2 \qquad (14)$$

when $\frac{\alpha}{\gamma} > \frac{\beta}{\eta}$, the variance of the stationary membrane potential (and hence also the output rate) increases by proportional increase of both inhibitory and excitatory rates and reaches a constant value $\frac{\alpha}{\gamma}$. This condition translates into $w_E(V_{Rexc} - \langle V_{st} \rangle)^2 > w_I(V_{Rinh} - \langle V_{st} \rangle)^2$. As long as $\langle V_{st} \rangle$ is adequately lower than V_{th}, $w_E(V_{Rexc} - \langle V_{st} \rangle) \approx w_I(V_{Rinh} - \langle V_{st} \rangle)$ and the condition mentioned above holds because $(V_{Rexc} - \langle V_{st} \rangle)$ is greater than $(V_{Rinh} - \langle V_{st} \rangle)$ by a factor of about 2.

We remind the reader that the Gaussian approximation is only legitimate in the case of small τ and for a low firing rate regime. We want to consider cases in which these two conditions are not satisfied. Firstly, at higher values of τ_{syn} and at the stationary values of the average membrane potential lower than the threshold (low firing regime), there is an inversion of the mentioned scenario. In this case, at sufficiently high values of the input rates, conditioned on constant average membrane potential, the variance of the membrane potential and accordingly the output rate decrease (Fig.4A). This is due to the filtering effect of the input by the gradual decay of the synaptic conductances. Using the method of τ-expansion of section 1.1.1, we will consider autocorrelation in synaptic conductance and partially take into account this effect. The filtering of the high-frequency signal by the slow conductances is a mechanism of gain control. In general, the decay and the rise time of the inhibitory synapses are longer than the excitatory ones which would highlight the inhibitory input as its overall strength increases through temporal persistence. In addition, the voltage-dependent inhibitory current is higher at higher values of the membrane potential. The balanced average membrane potential is somewhere in the mid-range. Longer synaptic decay time constant, higher synaptic strength, delay, and potential dependence of the inhibitory synapses increase the overall inhibition strength and compensate for the smaller number of them in comparison with excitatory synapses. It should be noted that output rates on the constant voltage level line with balanced input at $V = -57mv$ vary linearly with the input rates at moderate rate values corresponding to the low firing rate regime (Fig.4A).

On the other hand, when the stationary average membrane potential is located right at the threshold value, in conflict with the low firing regime assumption, equation 14 does not hold and higher rates of balanced input lead to a higher output rate independent of the value of τ_{syn} (Fig.4B). Moreover, the output rate varies like $\sqrt{I_{in}}$. For analyzing the low firing rate, we could linearize the output rate around the midpoint of the neuron potential range, i.e., at $V = -57mv$.

1.2 Condition for Poisson output

In the model of conductance based synaptic currents, the output spike train is Poisson in the regime of fluctuation driven spiking when the stationary average potential is away from the threshold. To analyze the condition for Poisson

firing, we investigate the case where the stationary membrane potential is below firing threshold. When excitatory and inhibitory currents are matched in a way that the stationary membrane potential V_{st} is close to the firing threshold V_{th}, the approximations in the previous section are no longer valid. In the limit of a high number of affarents the drift term would set the average membrane potential to its stationary value in a short time. Here we calculate the mean firing time and variance of it based on the approximation that fluctuations in the input close to the stationary average potential are weakly dependent on the voltage level.

Therefore, our problem is reduced to the well known problem of the first passage time of a Brownian particle evolving as $\frac{dx}{dt} = -kx + \xi(t)$ to reach the threshold a. ξ is white noise with variance σ. We want to approximate results for first passage time moments when the stationary membrane potential is close to the threshold. The goal here is to obtain approximate analytical results for firing rate and CV of the spike time interval to identify conditions for output Poisson firing and linearization of the output rate.

We apply the Siegert formula (Siegert (1951)) for the first passage time in our case. Let us take $x = v - \frac{a}{b}$, $x_0 = v_0 - \frac{a}{b}$ and $x_{th} = v_{th} - \frac{a}{b}$ where $\langle V \rangle_{st} = \frac{a}{b}$. Thus, the random variable x evolves as $\frac{dx}{dt} = -bx + \sigma(x)\xi(t)$. where ξ is a white noise with unit variance and

$$\sigma^2(x) = \frac{1}{C^2}\{D_e(v_{Rexc} - \frac{a}{b} + x)^2 + D_i(v_{Rinh} - \frac{a}{b} + x)^2)\}$$
$$b = \frac{1}{C}(g_{Leak} + g_0 w_e \lambda_e \tau + g_0 w_i \lambda_i \tau) \tag{15}$$

For the case of Poisson current to the cell, if the mean value is close to the threshold, the magnitude of fluctuations does not vary much in the interval $[V_{rest}, V_{th}]$, therefore in the following we neglect dependence of σ on x.

1.2.1 First passage time statistics

To determine first passage time(FPT) statistics let us first review the recursive formula for FPT moments as discussed in Siegert (1951). Suppose the stochastic process X_t with conditional probability density $P(x, t \mid x_0)$ satisfies the Fokker-Planck equation

$$\frac{\partial p(x,t \mid x_0)}{\partial t} = -\frac{\partial}{\partial x}[A(x)p(x,t \mid x_0)] + \frac{1}{2}\frac{\partial^2}{\partial x^2}(B(x)p(x,t \mid x_0))$$

with initial and boundary conditions $P(x, t_0 \mid x_0) = \delta(x - x_0)$ and $P(\pm\infty, t \mid x_0) = 0$, respectively. The first passage time probability density $\rho(\Theta \mid t, x_0)$ of

the stochastic process following the above Fokker-Planck equation satisfies

$$\rho(\Theta \mid t, s) = -2\frac{\partial}{\partial t}\int_{-\infty}^{s} P(\Theta, t \mid y)dy$$

Based on this equation we can write a recursion formula for moments of FPT with diffusion strength $B(y)$ and drift term $A(y)$:

$$t_n(\Theta \mid x_0) = n\int_{x_0}^{\Theta}\frac{2dz}{B(z)W(z)}\int_{-\infty}^{z} W(k)t_{n-1}(\Theta \mid k)dk \tag{16}$$

with $t_0 = 1$, and $W(k)$ is the stationary probability distribution

$$W(k) = \frac{C}{B(x)}exp[\int dx\frac{2A(x)}{B(x)}] \tag{17}$$

In particular, for the first moment we have

$$t_1(\Theta \mid x_0) = \int_{x_0}^{\Theta}\frac{2dz}{B(z)W(z)}\int_{-\infty}^{z} W(x)dx \tag{18}$$

For the case of Langevin equation in section 1.2, writing $x_0 = (V_{leak} - V_{th}) + (V_{th} - \frac{a}{b}) = -\Theta + x_{th}$ and using equations 17 and 18, the average first passage time can be written as

$$\begin{aligned}
t_1(x_{th} \mid x_0) =& \frac{\sqrt{\pi}}{b}\int_{x_0\sqrt{\frac{b}{\sigma^2}}}^{x_{th}\sqrt{\frac{b}{\sigma^2}}} e^{z^2}(1 + erf(z)) \\
=& \frac{\sqrt{\pi}}{b}[\int_{0}^{x_{th}\sqrt{\frac{b}{\sigma^2}}} e^{z^2}(1 + erf(z)) \\
&- \int_{0}^{(-\Theta + x_{th})\sqrt{\frac{b}{\sigma^2}}} e^{z^2}(1 + erf(z))]
\end{aligned}$$

where $erf(.)$ is the Gauss error function.

Taking $y = x_{th}\sqrt{\frac{b}{\sigma^2}}$ and $\theta = \Theta\sqrt{\frac{b}{\sigma^2}}$ and using the following series expansion for small y

$$\int_{0}^{y} e^{z^2}dz = y + \frac{y^3}{3} + \frac{y^5}{10}...$$

$$\int_0^y e^{z^2} erf(z)dz = \frac{1}{\sqrt{\pi}}(y^2 + \frac{y^4}{3} + \frac{4y^6}{45}...)$$
$$\int_0^{-\theta+y} e^{z^2}(1+erf(z))dz = \int_0^{-\theta} e^{z^2}(1+erf(z))dz + y(e^{-\theta^2}(1+erf(-\theta))$$
$$\approx \int_0^{-\theta} e^{z^2}(1+erf(z))dz \equiv \kappa \tag{19}$$

we arrive at the following approximation for the mean passage time,

$$t_1(x_{th} \mid x_0) = -\frac{\sqrt{\pi}}{b}\kappa + [(\frac{a}{b} - V_{rest})\sqrt{\frac{b}{\sigma^2}}]\frac{\sqrt{\pi}}{b} + \frac{\sqrt{\pi}}{b}[(V_{th} - \frac{a}{b})]\sqrt{\frac{b}{\sigma^2}} + \frac{1}{b}[(V_{th} - \frac{a}{b})\sqrt{\frac{b}{\sigma^2}}]^2 \tag{20}$$

It can be seen from equation 14 that the factor $\sqrt{\frac{b}{\sigma^2}}$ in large balanced rates of excitatory and inhibitory inputs asymptotically approaches a constant value. Therefore, we can approximate κ to be very weakly dependent on input rates and take it as a constant factor.

we can write down the average rate of firing, $\rho = \frac{1}{t_1(x_{th} \mid x_0)}$, in the case that the average stationary potential is close to threshold as

$$\rho = \frac{b}{\sqrt{\pi}}(\frac{1}{\kappa + (V_{th} - \frac{a}{b})\sqrt{\frac{b}{\sigma^2}}}) \tag{21}$$

In Figure 5, we have plotted this rate approximation which causes only a very small error when the average potential is near threshold. If $\langle v \rangle = \frac{a}{b}$ is constant for balanced inhibitory and excitatory input rates, then the output rate of the neuron would be linearly proportional to the input rates via the factor b. Moreover the output rate would decrease as $\frac{1}{x_{th}}$ with the increase of the distance of the stationary average potential from the threshold.

Next, we want to investigate the variance of first passage time. From the recursion formula 16 we have

$$t_2(x_{th} \mid x_0) = \frac{2\pi}{b^2}\int_{-\infty}^{x_{th}\sqrt{\frac{b}{\sigma^2}}} e^{z^2}(1+erf(z))^2 \int_z^{x_{th}\sqrt{\frac{b}{\sigma^2}}} dr e^{r^2}\Theta(r - x_0\sqrt{\frac{b}{\sigma^2}})$$

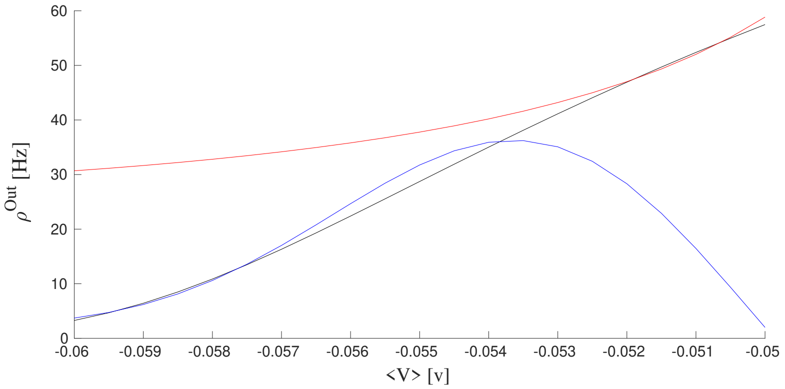

Fig. 5: Firing rate approximation by Gaussian assumption of Eq.12 in section 1 (blue) and by near-threshold high firing assumption(red) of Eq.21 are compared with simulation results (black curve). Here, we fix the inhibitory input and select a value of the excitatory input rate that leads to a specific mean stationary membrane potential shown on the x-axis.

After some straightforward calculations we arrive at

$$\begin{aligned} t_2(x_{th} \mid x_0) = & \frac{2\sqrt{\pi}}{b} t_1(x_{th} \mid x_0)[\int_0^{x_{th}\sqrt{\frac{b}{\sigma^2}}} e^{z^2}(1+erf(z))] \\ & + \frac{2\sqrt{\pi}}{b^2} ln(2)[\int_0^{x_{th}\sqrt{\frac{b}{\sigma^2}}} e^{z^2} - \int_0^{x_0\sqrt{\frac{b}{\sigma^2}}} e^{z^2}] \\ & - \frac{2\sqrt{\pi}}{b^2}[\phi(x_{th}\sqrt{\frac{b}{\sigma^2}}) - \phi(x_0\sqrt{\frac{b}{\sigma^2}})] \\ & - \frac{2\sqrt{\pi}}{b^2}[\psi(x_{th}\sqrt{\frac{b}{\sigma^2}}) - \psi(x_0\sqrt{\frac{b}{\sigma^2}})] \end{aligned}$$

where functions ϕ and ψ are multi-variable integrals containing powers of erf and e^{x^2} in the integrand with series expansion :

$$\phi(y) = \sum_{n=0}^{\infty} \frac{y^{2n+3}}{(n+1)!(2n+3)} \sum_{k=0}^{n} \frac{1}{2k+1}$$

$$\psi(y) = \sum_{n=0}^{\infty} \frac{2^n y^{2n+4}}{(n+2)(2n+3)!!} \sum_{k=0}^{n} \frac{1}{k+1}$$

Rewriting the first term in brackets in terms of first passage time and considering just terms linear in x_{th}

$$\begin{aligned} t_2(x_{th} \mid x_0) =& 2t_1(x_{th} \mid x_0)[t_1(x_{th} \mid x_0) \\ &+ \frac{\sqrt{\pi}}{b} \int_0^{(-\Theta+x_{th})\sqrt{\frac{b}{\sigma^2}}} e^{z^2}(1+erf(z))] \\ &+ \frac{2\sqrt{\pi}}{b^2} ln(2)[\int_0^{x_{th}\sqrt{\frac{b}{\sigma^2}}} e^{z^2} - \int_0^{x_0\sqrt{\frac{b}{\sigma^2}}} e^{z^2}] \\ &- \frac{2\sqrt{\pi}}{b^2}[\phi(x_{th}\sqrt{\frac{b}{\sigma^2}}) - \phi(x_0\sqrt{\frac{b}{\sigma^2}})] \\ &- \frac{2\sqrt{\pi}}{b^2}[\psi(x_{th}\sqrt{\frac{b}{\sigma^2}}) - \psi(x_0\sqrt{\frac{b}{\sigma^2}})] \\ \approx& 2t_1(x_{th} \mid x_0)^2 + \frac{2x_{th}\sqrt{\pi}}{b\sqrt{b}\sigma} ln(2) \\ &+ C(x_{th}=0) \end{aligned} \tag{22}$$

where the constant C, coming from the integral, is negative.

From the recursion formula (Eq.16)and Eq. 22,we end up with the following expression for the CV of the time interval between spikes:

$$CV^2 = \frac{Var(t)}{\langle t \rangle^2} \approx 1 + \frac{C}{t_1(x_{th} \mid x_0)^2} + 2\sqrt{\pi} ln(2) \frac{\frac{x_{th}}{b\sqrt{b}\sigma}}{t_1(x_{th} \mid x_0)^2} \tag{23}$$

where C is a negative constant (see Eq.22) and monotonically goes to zero as x_{th} increases. In the limit of large x_{th}, the second and the third term both go to zero and CV approaches 1. However, in the near threshold approximation the maximum of the third term occurs where CV is approaching 1. Expanding in powers of x_{th} , we arrive at

$$x_{th}^{opt} := V_{th} - \langle V \rangle_{st} = \frac{\pi\sigma}{2\sqrt{b}} \tag{24}$$

As shown in section 1.1.2 (Eq.14), $\frac{\sigma}{\sqrt{b}}$ reaches a constant value for high input rates. This can be used to determine the value of $\langle V \rangle_{st}$ that leads to maximal CV. At $V_P := \langle V \rangle_{st}^{opt} \approx -0.56mv$, the CV for different input rates has a maximum independently of the rate values.

2 Bifurcation analysis of a network with Sigmoid gain function

2.1 Location of the BT point

Here we want to approximate local bifurcation lines in the vontrol parameter space of a neuronal population with sigmoid gain function. At equilibrium, the population rates satisfy:

$$\begin{aligned} \rho_I &= g^I(\rho_I, c_{IE}\rho_E + c_{II}\rho_I + d\rho^I_{Ext}) - z_0 \\ \rho_E &= g^E(\rho_I, c_{EE}\rho_E + c_{EI}\rho_I + d\rho^E_{Ext}) - z_0 \end{aligned} \tag{25}$$

and the equation for the inhibitory nullcline can be written as :

$$\rho_E = \frac{1}{c_{IE}}\left(\frac{1}{k}[\log(\frac{(\rho_I + z_0)}{\rho_{max} - (\rho_I + z_0)}) + \log(\alpha)] - c_{II}\rho_I - d\rho^E_{Ext} - g_L\right) \tag{26}$$

The condition on zero trace $Tr(J) = J_{11} + J_{22} = 0$ of the Jacobian (Eq.31 main text) parameterized by the inhibitory nullcline curve(Eq.26) determines the value for w_{EE} at which a Hopf bifurcation can occur. Ignoring terms related to α, which are relatively small, from equating the trace to zero, we have

$$c^H_{EE} = \frac{2 - c_{II} * \rho_I(1 - \frac{\rho_I}{\rho_{max}})}{\rho_E(1 - \frac{\rho_E}{\rho_{max}})} \tag{27}$$

Then we have $w^H_{EE} = \frac{c^H_{EE}}{ck_{EE}(V_{RExc} - V_{th})}$. For each (ρ_E, ρ_I) point on the inhibitory nullcline (Eq.26), the above equation gives a value of w_{EE} which sets the trace of the Jacobian to zero at this point. The second equation in Eq.25 which corresponds to the excitatory nullcline determines ρ^E_{Ext} parameterized by ρ_{Inh}. Next, we should check the positivity of the determinant to sketch the Hopf bifurcation line in the $w_{EE} - \rho^E_{Ext}$ plane. Neglecting non-linearities caused by α, the determinant of the Jacobian conditioned on zero trace is

$$det(J)_{|Tr(J)=0} = -(1 - c_{II}\rho_I(1 - \frac{\rho_I}{\rho_{max}}))^2 - c_{IE}c_{EI}\rho_I(1 - \frac{\rho_I}{\rho_{max}})\rho_E(1 - \frac{\rho_E}{\rho_{max}}) \tag{28}$$

At extremely low values of the rates (near zero), the determinant is negative because of the -1 in the above formula. Conditioned on a sufficient amount of inhibitory feedback strength, which is proportional to $|c_{IE}c_{EI}|$, the determinant becomes positive at some point and the low fixed point loses stability

through a Hopf bifurcation. A point where both determinant and trace of J are zero, is called a *Bogdanov-Takens* (BT) *bifurcation point.*

Inserting $\rho_E(1-\frac{\rho_E}{\rho_{max}})$ from $det(J)_{|Tr(J)=0}=0$ into the denominator of the formula for w^H_{EE} and introducing the parameter $\gamma=\rho_I(1-\frac{\rho_I}{\rho_{max}})$, at the BT point in the low rate regime:

$$c^{BT}_{EE}=\frac{\gamma c_{IE}c_{EI}(2-c_{II}*\gamma)}{(1-c_{II}\gamma)^2} \tag{29}$$

When $\mid c_{II}\gamma \mid$ is at a moderate value, i.e. sufficiently greater than one :

$$c^{BT}_{EE}\approx\frac{c_{IE}c_{EI}}{c_{II}} \tag{30}$$

If we take number of connections to satisfy $\frac{k_{EI}}{k_{EE}}=\frac{k_{II}}{k_{IE}}$, then $w^{BT}_{EE}=\frac{w_{IE}w_{EI}}{w_{II}}$. On the semi-linear part of the inhibitory nullcline we have an approximate relation between rates of the form $\frac{\rho_I}{\rho_E}\approx\frac{w_{IE}}{w_{II}}$. The determinant on the line of zero trace when $\rho_E=\frac{w_{II}}{w_{IE}}:=\beta\rho_I$ is

$$det(J)_{|Tr(J)=0}=-1+2c_{II}\gamma+(c^2_{ii}-\beta c_{IE}c_{EI})\gamma^2 \tag{31}$$

The function $\gamma(\rho_I)$ has a maximum at $\frac{\rho_{max}}{2}$. With this in mind, the condition for positive determinant at a potential Hopf bifurcation fixed point at lower rates of the linear regime is

$$det(J)^L_{|Tr(J)=0}\approx-c^2_{II}\rho^2_I-c_{IE}c_{EI}\rho_E\rho_I|c_{II}|\rho^2_I(|c_{EI}|-|c_{II}|)>0 \tag{32}$$

Therefore, if $|c_{EI}|>|c_{II}|$, the Hopf bifurcation line survives in the linear regime. On this line, $w^{Hopf}_{EE}\approx\frac{2-c_{II}\gamma}{c^0_{EE}\beta\gamma}$, which has negative derivative $\frac{-2}{\beta\gamma^2}$. Thus, Hopf bifurcation in the linear regime occurs at lower values of w_{EE} compared to w^{BT}_{EE} . On the other hand, ρ^E_{Ext} should increase to satisfy the fixed point condition of equation 25 for the excitatory rate. Altogether, in the $(w_{EE}-\rho^E_{Ext})$ plane, the Hopf bifurcation line extends to lower w_{EE} and higher ρ^E_{Ext} from the low BT to high BT.

Ascending on the inhibitory nullcline, we reach the nonlinear high branch of the curve, where a linear relation between rates no longer holds and the second derivative of $\rho^*_E(\rho_I)$ will increase. In addition, $\gamma(\rho_{inh})$ decreases towards zero. Taking into account these two facts, on the high branch $det(J)^L_{|Tr(J)=0}$ decreases and passes through zero at another Bogdanov Takens point at high rate values. If inhibitory feedback is not strong enough, the conditions for Hopf bifurcation are not satisfied.

To sketch the saddle-node bifurcation line we should look at solutions to $det(J) = 0$. Inserting $\rho_E(\rho_I)$ from Equation 26 into $det(J)$, for each point on the inhibitory nullcline, there exists some w_{EE} for which $det(J) = 0$. The only condition to check is $w_{EE} > 0$. Again the condition that the excitatory nullcline intersects the inhibitory one at the fixed point determines ρ_{Ext}. Along the semi-linear section of the nullcline, the condition $det(J) = 0$ translates into the alignment of the slopes of the linearized nullclines. Therefore, along this section w_{EE} varies very little.

2.2 Normal form of BT at the low firing rate regime

Consider the system in the vicinity of the BT bifurcation point

$$\frac{dz}{dt} = f(z, \mu) = Jz + F(z), z, \mu \in R^2 \tag{33}$$

Suppose that at $\mu = 0$, the system has a fixed point at z_0 with a Jacobian with a zero eigenvalue of multiplicity two. At the BT point, there exist two generalized eigenvectors q_0 and q_1 such that

$$Jq_0 = 0, Jq_1 = q_0$$

Also for J^T we select vectors $p_{0,1}$ by

$$J^T p_1 = 0, J^T p_0 = p_1$$

with normalization

$$\langle p_0, q_0\rangle = \langle p_1, q_1\rangle = 1$$
$$\langle p_0, q_1\rangle = \langle p_1, q_0\rangle = 0$$

By linear change of coordinates with transformation matrix $T = (q_0, q_1)$, i.e., $x = Tz$, our system can be written as

$$\begin{pmatrix} \dot{x_1} \\ \dot{x_2} \end{pmatrix} = \begin{pmatrix} 0 & 1 \\ 0 & 0 \end{pmatrix} \begin{pmatrix} x_1 \\ x_2 \end{pmatrix} + \begin{pmatrix} f(x_1, x_2) \\ g(x_1, x_2) \end{pmatrix} \tag{34}$$

Introducing a direction-preserving time reparametrization and smooth invertible parameter changes, we can tranform the system to the normal form:

$$\begin{aligned} \frac{y_1}{d\tau} &= y_2 \\ \frac{y_2}{d\tau} &= \epsilon_1 + \epsilon_2 y_1 + a_2 y_1^2 + b_2 y_1 y_2 + O(y_1 {y_2}^3) \end{aligned} \tag{35}$$

$\epsilon_{1,2}(\mu)$ are transformed bifurcation parameters , $a_2 = g_{xx}/2$ and $b_2 = g_{xy} + f_{xx}$ and $dt = (1 + \Theta x_1)d\tau$. The y coordinates relate to the original $z = T^{-1}x$

coordinates via :

$$\begin{aligned} u_1 &= x_1, & y_1 &= u_1 \\ u_2 &= \dot{x}_1, & y_2 &= u_2 + \Theta u_1 u_2 \end{aligned} \tag{36}$$

where $\Theta = g_{yy} + 2f_{xy}$. Fixed points of the normal form of Equation 35 are $(y_1, y_2) = (\pm\sqrt{(\frac{-\epsilon_1}{a_2}}, 0)$. Taking $a_2 > 0$, when $\epsilon_1 < 0$, there exist two fixed points, with Jacobian

$$\begin{pmatrix} 0 & 1 \\ \pm 2\sqrt{\frac{-\epsilon_1}{a_2}} & \epsilon_2 \pm b_2\sqrt{\frac{-\epsilon_1}{a_2}} \end{pmatrix}$$

y_0^+ is a saddle in $\epsilon_1 < 0$ for all ϵ_2, while y_0^- is a sink for $\epsilon_2 < b_2\sqrt{\frac{-\epsilon_1}{a_2}}$ and a source for $\epsilon_2 > b_2\sqrt{\frac{-\epsilon_1}{a_2}}$. When $b_2 > 0$, then the line $\epsilon_2 = b_2\sqrt{\frac{-\epsilon_1}{a_2}}$ is a sub-critical Hopf bifurcation and when $b_2 > 0$ the same line is a supercritical Hopf bifurcation. To summarize, defining $\sigma = sgn(a_2 * b_2)$, if σ is negative then a stable limit cycle appears and the Hopf bifurcation is supercritical (Fig.**??**B1), but if σ is positive, we have a sub-critical Hopf bifurcation (Fig.**??**B2). As shown in Figure **??**, near the BT point apart from local bifurcations, i.e., Hopf and saddle-node, there is saddle-node separatrix loop bifurcation which annihilates the stable or unstable limit cycle that is produced by a super- or sub-critical Hopf bifurcation, respectively.

By writing the normal form we can analyze the type of BT from explicit linearization. For the case of J_{BT} in equation **??**, generalized eigenvectors are $q_0 = \begin{pmatrix} 1 & \alpha/\beta \end{pmatrix}$, $q_1 = \begin{pmatrix} 1 & (\alpha-1)/\beta \end{pmatrix}$, $p_1 = (1, -\beta/\alpha)$ and $p_0 = (1/\alpha - 1, \beta/\alpha)$. Therefore, new parameterized coordinates are $x_1 = E/\beta - \alpha/\beta(E - I)$ and $x_2 = E - I$. The normal form parameters a_2 and b_2 are

$$\begin{aligned} a_2 &= \frac{1}{2} < p_1, F(q_0, q_0) > \\ b_2 &= < p_0, F(q_0, q_0) > + < p_1, F(q_0, q_1) > \end{aligned} \tag{37}$$

where $F^1(q_0, q_i) = \sum_{l,k} f_{lk} q_0^l q_i^k$ and $F^2(q_0, q_i) = \sum_{l,k} g_{lk} q_0^l q_i^k$. Using the logistic gain function, second derivatives in $E - I$ coordinates can be written as

$$\begin{aligned} g_{EE} &\propto W_{IE}^2 g_I(1 - g_I)(1 - 2g_I) \\ g_{EI} &\propto -|W_{II}||W_{IE}| g_I(1 - g_I)(1 - 2g_I) \\ f_{EE} &\propto W_{EE}^2 f_E(1 - f_E)(1 - 2f_E) \end{aligned}$$

where $g_{EI} < 0$, $f_{EE} > 0$ and $g_{EE} > 0$. After straightforward but lengthy calculation we can confirm $sgn(\sigma) = sgn(a_2)sgn(b_2) < 0$ using $|W_{II}|g_I(1 - g_I) = W_{EE}f_E(1 - f_E)$ and $\frac{\beta}{\alpha} = \frac{c_{EI}}{c_{EE}} = \frac{c_{II}}{c_{IE}}$ at the lower BT point.

References

Siegert A (1951) On the first passage time probability problem. Phys Rev 81. https://doi.org/10.1103/PhysRev.81.617
